# A glycyl radical enzyme enables hydrogen sulfide production by the human intestinal bacterium *Bilophila wadsworthia*

**DOI:** 10.1073/pnas.1815661116

**Published:** 2019-02-04

**Authors:** Spencer C. Peck, Karin Denger, Anna Burrichter, Stephania M. Irwin, Emily P. Balskus, David Schleheck

**Affiliations:** ^a^Department of Chemistry and Chemical Biology, Harvard University, Cambridge, MA 02138;; ^b^Department of Biology, University of Konstanz, D-78457 Konstanz, Germany;; ^c^Konstanz Research School Chemical Biology, University of Konstanz, D-78457 Konstanz, Germany

**Keywords:** carbon-sulfur bond-cleaving glycyl radical enzyme, human gut microbiome, anaerobic degradation, organosulfonate respiration, human health

## Abstract

Hydrogen sulfide (H_2_S) production in the intestinal microbiota has many contributions to human health and disease. An important source of H_2_S in the human gut is anaerobic respiration of sulfite released from the abundant dietary and host-derived organic sulfonate substrate in the gut, taurine (2-aminoethanesulfonate). However, the enzymes that allow intestinal bacteria to access sulfite from taurine have not yet been identified. Here we decipher the complete taurine desulfonation pathway in *Bilophila wadsworthia* 3.1.6 using differential proteomics, in vitro reconstruction with heterologously produced enzymes, and identification of critical intermediates. An initial deamination of taurine to sulfoacetaldehyde by a known taurine:pyruvate aminotransferase is followed, unexpectedly, by reduction of sulfoacetaldehyde to isethionate (2-hydroxyethanesulfonate) by an NADH-dependent reductase. Isethionate is then cleaved to sulfite and acetaldehyde by a previously uncharacterized glycyl radical enzyme (GRE), isethionate sulfite-lyase (IslA). The acetaldehyde produced is oxidized to acetyl-CoA by a dehydrogenase, and the sulfite is reduced to H_2_S by dissimilatory sulfite reductase. This unique GRE is also found in *Desulfovibrio desulfuricans* DSM642 and *Desulfovibrio alaskensis* G20, which use isethionate but not taurine; corresponding knockout mutants of *D. alaskensis* G20 did not grow with isethionate as the terminal electron acceptor. In conclusion, the novel radical-based C-S bond-cleavage reaction catalyzed by IslA diversifies the known repertoire of GRE superfamily enzymes and enables the energy metabolism of *B. wadsworthia*. This GRE is widely distributed in gut bacterial genomes and may represent a novel target for control of intestinal H_2_S production.

## Significance

This paper describes a pathway for anaerobic bacterial metabolism of taurine (2-aminoethanesulfonate), an abundant substrate in the human intestinal microbiota, by the intestinal bacterium and opportunistic pathogen, *Bilophila wadsworthia*. This metabolism converts taurine to the toxic metabolite hydrogen sulfide (H_2_S), an activity associated with inflammatory bowel disease and colorectal cancer. A critical enzyme in this pathway is isethionate sulfite-lyase, a member of the glycyl radical enzyme family. This enzyme catalyzes a novel, radical-based C-S bond-cleavage reaction to convert isethionate (2-hydroxyethanesulfonate) to sulfite and acetaldehyde. This discovery improves our understanding of H_2_S production in the human body and may also offer new approaches for controlling intestinal H_2_S production and *B. wadsworthia* infections.

The metabolism of dietary and host-derived sulfur-containing compounds to hydrogen sulfide (H_2_S) by members of the human gut microbiota has many prominent connections to host health and disease (1, 2). H_2_S is a potent genotoxin and may contribute to the onset of colorectal cancer (3). This metabolite can also reduce disulfide bonds in the mucus layer of the gut epithelium, disrupting its barrier function and potentially playing a role in inflammatory bowel disease (IBD) (4). As H_2_S can induce antibiotic resistance, production of this compound in the gut microbiota may trigger blooms of opportunistic bacteria during antibiotic treatment (5). However, H_2_S produced by gut microbes may also act as a signaling molecule in the host, potentially leading to beneficial effects, such as cardioprotection (2, 6). Elucidating the influence of gut microbial sulfur metabolism and H_2_S production on host health and disease represents an important unresolved challenge in human microbiota research (1, 2, 7, 8).

**Fig. 1.**
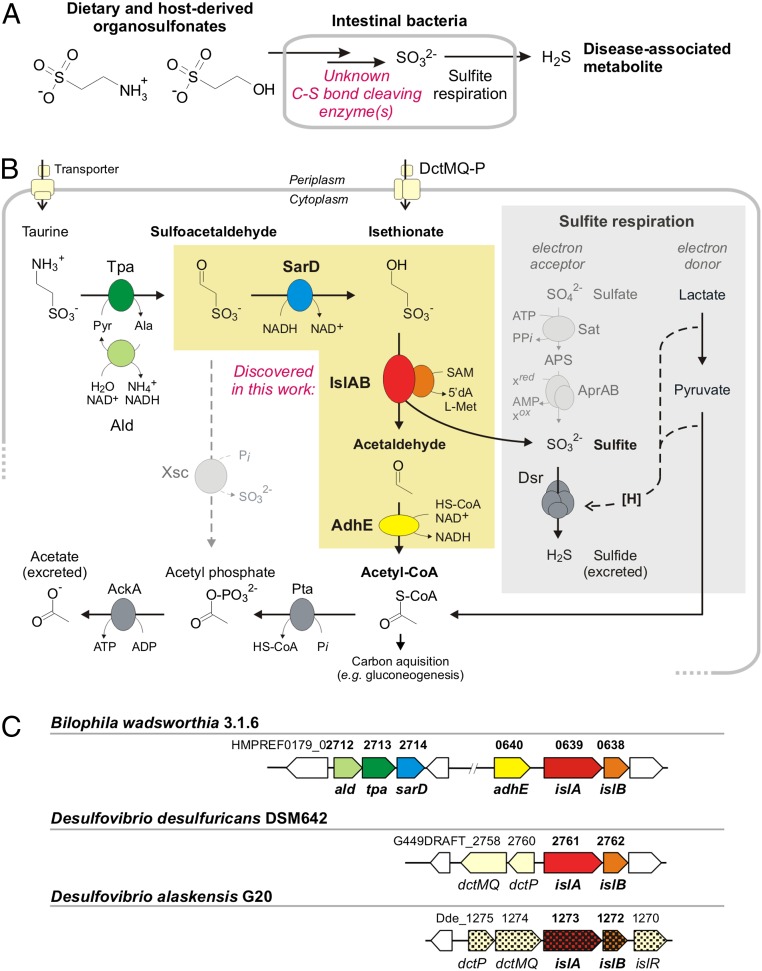
Metabolism of taurine and isethionate by the human gut bacterium *B. wadsworthia* and by *Desulfovibrio* spp. (*A*) *B. wadsworthia* and other intestinal bacteria degrade dietary and host-derived organosulfonates to access sulfite as an electron acceptor for their anaerobic respiration. The desulfonation reaction in *B. wadsworthia* has not yet been identified. (*B*) Summary of the pathways investigated in this study (no single organism represented). *B. wadsworthia* uses taurine and isethionate as electron acceptors, and the two *Desulfovibrio* spp. strains can use isethionate, but not taurine. All three strains lack the Xsc enzyme known in many aerobic bacteria (dashed line on the left). Instead, the GRE IslA with its GRE-activase component (IslB) are found in both *B. wadsworthia* and the *Desulfovibrio* spp., and a novel NADH-coupled, isethionate-forming sulfoacetaldehyde reductase (SarD) is found only in *B. wadsworthia*. The sulfite released by the GRE is reduced to sulfide by Dsr and coupled to proton translocation for ATP synthesis (symbolized as [H]) when using electrons from oxidation of an alternative electron donor, such as lactate (gray box). AckA, acetate kinase; Pta, phosphotransacetylase. (*C*) The gene clusters identified in this study. In *B. wadsworthia*, two separate clusters encode for taurine transport and conversion to isethionate (*ald*, *tpa*, and *sarD*), and also for isethionate desulfonation and the conversion of acetaldehyde to acetyl-CoA (*adhE*, *islA*, and *islB*). The *Desulfovibrio* strains harbor only a gene cluster for isethionate transport (*dctP* and fused *dctMQ*) and desulfonation (*islA* and *islB*), and acetaldehyde dehydrogenases are encoded elsewhere in their genomes (not depicted in *C*). Single-gene knockouts in *D. alaskensis* G20 that result in an inability to use isethionate (but not free sulfite) are indicated by squared gene symbols.

Significant gaps remain in our understanding of the gut microbial metabolic pathways and enzymes that generate H_2_S (i.e., sulfidogenesis). For example, the human gut bacterium and opportunistic pathogen *Bilophila wadsworthia* (9) produces H_2_S when respiring (bi)sulfite (HSO_3_^−^) released from organosulfonate substrates (R_3_C-SO_3_^–^), including the abundant dietary and host-derived molecule taurine (2-aminoethanesulfonate), as well as isethionate (2-hydroxyethanesulfonate) (Fig. 1*A*) (10). Although *B. wadsworthia* is typically present in low abundance in the colonic microbiota of healthy humans (11), it has been associated with appendicitis and bone, brain, liver, and ear abscesses (12), as well as with colorectal cancer (13). In mice, *B. wadsworthia* can cause systemic inflammation (14) and proliferates concomitantly with the onset of ulcerative colitis when taurine-conjugated bile acids are more abundant in the gastrointestinal tract (15).

Metabolism of taurine and isethionate by *B. wadsworthia* involves a desulfonation reaction (Fig. 1 *A* and *B*) to generate free sulfite, which is then reduced to H_2_S via the action of dissimilatory sulfite reductase (Dsr; desulfoviridin) for energy conservation by anaerobic sulfite respiration (10, 16). However, the enzymes involved in this critical C-S bond-cleaving step have not yet been identified in *B. wadsworthia*. The known metabolic pathways for taurine and isethionate, deciphered almost exclusively in aerobic bacteria, are diverse but invariably involve sulfoacetaldehyde as the critical intermediate (17) (see also MetaCyc pathways taurine degradation I–IV). In these bacteria, sulfoacetaldehyde is desulfonated by the oxygen-insensitive, thiamine-diphosphate (TDP)-dependent sulfoacetaldehyde acetyltransferase (Xsc) (18, 19) to produce acetyl phosphate, which is used to generate ATP or acetyl-CoA (Fig. 1*B*), and sulfite, which is then oxidized to sulfate by sulfite dehydrogenase in most aerobic bacteria (not depicted in Fig. 1*B*). Notably, the sulfoacetaldehyde-desulfonating Xsc enzyme is also used by strictly anaerobic *Desulfonispora thiosulfatigenes* GKNTAU, which ferments taurine to acetate, ammonium, and thiosulfate (19).

Although an analogous C-S bond-cleaving reaction has been proposed to operate in the strictly anaerobic, taurine- and isethionate-respiring, sulfidogenic bacterium *B. wadsworthia*, no valid Xsc homologs can be detected in the genomes of these organisms; that is, all homologs are annotated as catalytic, TDP-binding subunits of acetolactate synthase genes and are coencoded with their small-subunit genes (<33% amino acid sequence identity). Furthermore, no sulfoacetaldehyde-desulfonating Xsc enzyme activity can be observed in cell-free extracts of taurine-grown cells (20).

In the present study, we first investigated taurine and isethionate catabolism in the Human Microbiome Project (HMP) reference strain *Bilophila wadsworthia* 3.1.6 (21), and then examined isethionate catabolism in *Desulfovibrio desulfuricans* subsp. *desulfuricans* DSM642 (16) and *Desulfovibrio alaskensis* G20 (22), once we realized that these two pathways involve the same type of oxygen-sensitive desulfonation reaction. *Desulfovibrio* spp. are sulfate-reducing bacteria also found in the human intestinal microbiota (11, 23) and these organisms are also of clinical importance, associated with abdominal infections and bacteremia (24).

Because taurine and isethionate catabolism are inducible in these bacteria, we adopted a differential-proteomics and reverse-genetics approach (25, 26) to identify the complete organosulfonate catabolic pathways.

## Results

### Proteomic Experiments Reveal Putative Desulfonating Glycyl Radical Enzymes in *Bilophila* and *Desulfovibrio* Strains.

**Fig. 2.**
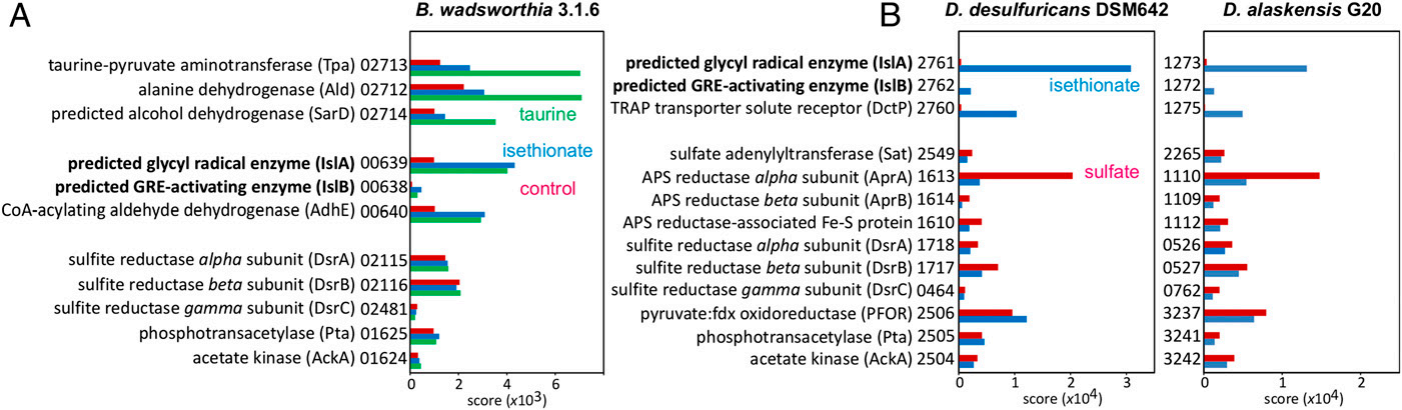
Proteomics experiments with cell-free extracts reveal a putative desulfonating GRE. (*A* and *B*) Proteins encoded by the taurine and isethionate utilization gene clusters, including the predicted isethionate GRE (IslA) and GRE-activase (IslB) components, are specifically and strongly expressed during growth with taurine (*B. wadsworthia*) and isethionate (*B. wadsworthia*, *Desulfovibrio* strains). Constitutively expressed proteins for, for example, sulfite reduction (Dsr) and metabolism of acetyl-CoA (Pta and AckA), are shown for comparison. IMG locus tag numbers are given; their prefixes are shown in Fig. 1*C*. Shown are results of representative total proteomic analyses replicated at least twice with extracts prepared from independent growth experiments.

We initially investigated the missing desulfonation step in taurine and isethionate catabolism using the HMP reference isolate *B. wadsworthia* 3.1.6 (21). Strain 3.1.6 was grown in media containing either taurine or isethionate as an electron acceptor. Proteomics experiments implicated two gene clusters in taurine catabolism (Fig. 2*A*), one of which was also linked to isethionate catabolism (Fig. 1*C*). These gene clusters are conserved in all sequenced *Bilophila* genomes. The first gene cluster encodes the known enzymes taurine:pyruvate aminotransferase (Tpa) and alanine dehydrogenase (Ald), as previously identified in a different *B. wadsworthia* isolate (27, 28). Tpa converts taurine to sulfoacetaldehyde, and Ald regenerates the amino group acceptor for Tpa (Fig. 1*B*). Both proteins were detected with a high relative abundance (i.e., with a higher protein score and hit rank) in taurine-grown cells, but not in isethionate-grown cells (Fig. 2*A*). Another protein encoded in this gene cluster, a predicted iron-dependent alcohol dehydrogenase (SarD in Fig. 1), was also detected in higher relative abundance specifically in taurine-grown cells but had not been previously characterized. Surprisingly, the second gene cluster encodes a glycyl radical enzyme (GRE) and its cognate radical *S*-adenosylmethionine (SAM)-activating enzyme (Fig. 1*C*). These proteins were consistently detected at much higher relative abundance (and with the highest hit rank) in both taurine-grown and isethionate-grown cells (Fig. 2*A*). Another protein encoded directly upstream of the GRE was also detected in higher relative abundance in both taurine-grown and isethionate-grown cells (Fig. 2*A*), a predicted CoA-acylating aldehyde dehydrogenase (AdhE) (Fig. 1*C*).

The discovery of these gene clusters allowed us to formulate a biochemical hypothesis for taurine catabolism in *B. wadsworthia*. The initial deamination of taurine would generate sulfoacetaldehyde (Fig. 1*B*). Rather than undergoing C-S bond cleavage by an Xsc-type enzyme, we hypothesized that sulfoacetaldehyde instead could be reduced to isethionate by the coinduced alcohol dehydrogenase (*sarD*) encoded in the *tpa-ald* gene cluster. We then envisioned that the GRE from the second gene cluster might catalyze the conversion of isethionate to acetaldehyde and sulfite.

GREs are extremely oxygen-sensitive enzymes that use protein-centered radical intermediates in catalysis, with the key glycine-centered radical installed posttranslationally by a partner activating enzyme that is a member of the radical SAM enzyme family (*SI Appendix*, Fig. S1*A*). GREs catalyze challenging biochemical transformations that play important roles in primary metabolism under strictly anoxic conditions, including a range of C-C, C-O, and C-N bond-cleavage reactions (29). Although C-S bond cleavage had not been reported for any GRE, the amino acid sequence of the identified *Bilophila* GRE resembles the sequences of the C-O bond-cleaving GRE 1,2-propanediol dehydratase from *Roseburia inulinivorans* (32% identity), which converts 1,2-propanediol to water and propionaldehyde (propanal) (30), and the C-N bond-cleaving GRE choline trimethylamine-lyase (CutC) from *D. alaskensis* G20 (31% identity), which metabolizes choline to trimethylamine and acetaldehyde (31). This relationship suggests the potential for the *B. wadsworthia* GRE to function as a C-S bond-cleaving isethionate sulfite-lyase (IslA) of a desulfonation pathway for taurine (Fig. 1*B*). The acetaldehyde produced could then be further metabolized to acetyl-CoA by the CoA-acylating aldehyde dehydrogenases (*adhE*) encoded in the GRE gene cluster (Fig. 1 *B* and *C*).

To obtain further evidence for this biochemical hypothesis and the role of the putative isethionate metabolizing GRE, we examined the genomes and proteomes of two additional bacteria that use isethionate (but not taurine) as a terminal electron acceptor and, like *B. wadsworthia*, do not encode an Xsc homolog: *Desulfovibrio desulfuricans* subsp. *desulfuricans* DSM642 (16) and *D. alaskensis* G20 (22). Using an analogous differential-proteomics and reverse-genetics approach, we identified similar enzymatic machinery in these organisms that were specifically produced during growth with isethionate. A candidate GRE and GRE-activating enzyme were detected in high abundance in cells grown with isethionate but not in control cells (Fig. 2*B*). The sequences of these enzymes strongly resembled the corresponding GRE and activase from *B. wadsworthia* (pairwise identities of >73% and >52%, respectively). A protein encoded next to the putative isethionate-metabolizing GRE/activase was also detected in high abundance only in isethionate-grown cells (Fig. 2*B*). This predicted substrate-binding protein (DctP) of a tripartite ATP-independent periplasmic transporter (Fig. 1*B*) may be involved in isethionate transport (*SI Appendix*, Fig. S2). We found highly similar gene clusters in the genomes of a wide range of sulfidogenic *Desulfovibrio* spp., including *Desulfovibrio fairfieldensis*, *Desulfovibrio piger*, *Desulfovibrio termitidis*, *Desulfovibrio vulgaris*, and *Desulfovibrio ruminis*, as well as in the genomes of many *Desulfitobacterium*, *Desulfomicrobium*, and *Desulfotomaculum* strains (see below). The presence of the putative C-S bond-cleaving GRE in these organisms strongly implicates this enzyme in isethionate metabolism.

### Activities of Cell-Free Extracts and Genetic Knockouts Confirm the Proposed Organosulfonate Catabolic Pathways.

We next sought to further verify these proposed taurine and isethionate catabolic pathways by examining cell-free extracts of *B. wadsworthia* and *Desulfovibrio* spp. for the newly postulated enzyme activities. First, high specific activity of an NADH-coupled sulfoacetaldehyde reductase was detected in extracts of taurine-grown *B. wadsworthia* cells (mean ± SD, 1.2 ± 0.12 U/mg; *n* = 5), with much less activity observed in extracts of isethionate-grown cells (<0.5 U/mg). Second, formation of sulfite and acetaldehyde, the predicted products of the isethionate sulfite-lyase reaction, was detected in extracts of the two *Desulfovibrio* strains when grown with isethionate only in the presence of isethionate as substrate. This activity—6.6 and 3.8 mU/mg for strains DSM642 and G20, respectively, calculated from the rate of sulfite formation and for representative reactions shown in *SI Appendix*, Fig. S3—was observed only when great care was taken to perform the cell harvest, cell extract preparation, and the enzyme assays under strictly anoxic conditions in presence of a strong reducing agent, titanium(III) nitrilotriacetate (*SI Appendix*, *Materials and Methods*). The air sensitivity of this activity is consistent with involvement of a GRE. Strictly anoxic extracts of taurine-grown *B. wadsworthia* cells also exhibited sulfite formation when exposed to isethionate as substrate (5.3 mU/mg for a representative reaction shown in *SI Appendix*, Fig. S3), indicative of a desulfonation reaction, but we were not able to detect acetaldehyde formation in these extracts (see below). When isethionate was replaced with taurine as a potential substrate for the GRE, no sulfite formation was observed. We also observed no sulfite formation in reactions additionally containing the cosubstrates needed to generate isethionate from taurine (pyruvate and NADH) in *B. wadsworthia* (Fig. 1*B*). In summary, we confirmed an oxygen-sensitive IslA activity in extracts of the two isethionate-grown *Desulfovibrio* strains and in taurine-grown *B. wadsworthia*, but we were not able to demonstrate desulfonation of taurine by successfully coupling all three enzymes of *B. wadsworthia* (Tpa, sulfoacetaldehyde reductase, and putative GRE) in cell-free extract under the conditions tested.

In addition, we confirmed that the genes encoding the putative isethionate-metabolizing GRE, activase, and other proteins encoded in the gene cluster are required for isethionate utilization by examining single-deletion strains from a *D. alaskensis* G20 transposon library (22). While wild-type and mutant strains grew equally well in media containing sulfite as the sole electron acceptor, knockout of any of the genes in the putative isethionate utilization gene cluster (GRE; activase or transporter genes) or of a predicted regulator gene (*islR*) (Fig. 1*C*) abolished growth with isethionate (*SI Appendix*, Fig. S4), confirming that these genes are essential for the use of isethionate-derived sulfite, but not free sulfite, as an electron acceptor.

### In Vitro Reconstruction of Taurine Catabolism.

Having identified the complete pathways for taurine and isethionate metabolism in these strictly anaerobic bacteria, we sought to characterize the key enzymes from these processes in vitro. We first cloned and heterologously expressed the genes from the two *B. wadsworthia* gene clusters in *Escherichia coli* and purified the enzymes (*SI Appendix*, Fig. S5). We verified that aminotransferase Tpa catalyzes the deamination of taurine with pyruvate to generate sulfoacetaldehyde and alanine (28). We then confirmed that the putative sulfoacetaldehyde reductase SarD reduces sulfoacetaldehyde to isethionate in an NADH-dependent manner (*SI Appendix*, Figs. S6 and S7). SarD did not accept acetaldehyde as a substrate. Combining Tpa and SarD resulted in the conversion of taurine to isethionate via sulfoacetaldehyde as confirmed by LC-MS (*SI Appendix*, Fig. S8). Taken together, these results confirm that the SarD enzyme provides the link between taurine and isethionate catabolism in *B. wadsworthia*.

**Fig. 3.**
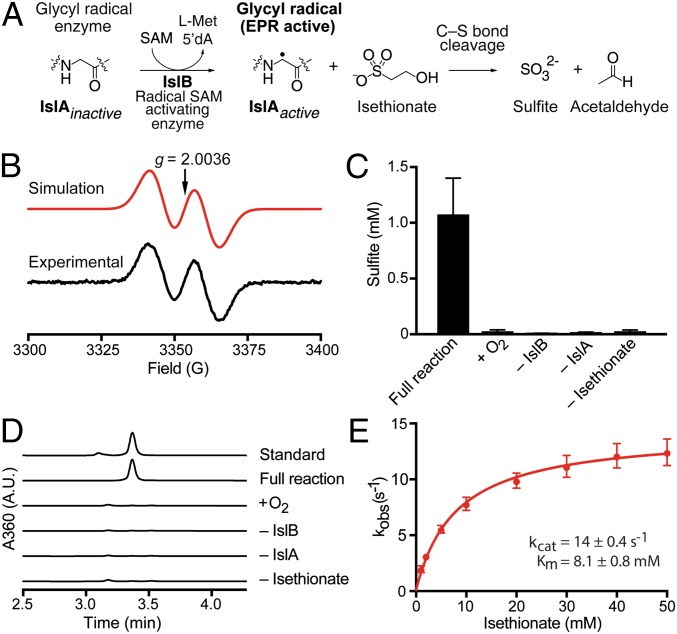
The *B. wadsworthia* GRE is a C-S bond-cleaving IslA. (*A*) The GRE-activating radical SAM enzyme IslB installs a protein-centered, stable glycyl radical within the GRE IslA. Activated IslA then catalyzes a C-S bond-cleavage reaction with isethionate as the substrate. The mechanistic hypothesis is illustrated in *SI Appendix*, Fig. S1*B*. (*B*) Electron paramagnetic resonance spectroscopy demonstrating glycyl radical formation on recombinantly produced and purified IslA after its activation by recombinant purified and reconstituted GRE-activase IslB. (*C*) Conversion of isethionate to sulfite by IslA was observed only in the absence of molecular oxygen and in the presence of all reaction components, as was acetaldehyde formation (*D*). The data in *C* are shown as the mean ± SD of three technical replicates, and data in *D* are shown as representative HPLC chromatograms of a minimum of two technical replicates. (*E*) The Michaelis–Menten kinetics of isethionate cleavage were determined by using a coupled spectrophotometric assay with yeast alcohol dehydrogenase and NADH. The *k*_cat_ was 14 ± 0.4 s^−1^, and the *K*_m_ was 8.1 ± 0.8 mM. Data are shown as the mean ± SD of four technical replicates.

We then investigated the proposed C-S–cleaving GRE that generates sulfite, isethionate sulfite-lyase (IslA). The *B. wadsworthia* GRE-activating enzyme (IslB) was expressed, purified, and characterized under anoxic conditions, whereas the *B. wadsworthia* IslA was expressed and isolated under oxic conditions and then rendered anoxic (*SI Appendix, Material and Methods*). The purified activase IslB was reconstituted by reduction with dithionite (*SI Appendix*, Fig. S9) and then incubated with the purified GRE IslA in the presence of SAM and acriflavine as a photosensitizer. This installed the glycyl radical on 13 ± 0.1% (mean ± SD of three replicates) of all GRE polypeptides as determined by electron paramagnetic resonance spectroscopy (Fig. 3*B*). Incubation of the activated IslA with isethionate resulted in substrate consumption (*SI Appendix*, Fig. S10) and formation of sulfite (Fig. 3*C*) and acetaldehyde (Fig. 3*D*). No reaction was observed when oxygen was present or when the activase, GRE, or the substrate was omitted (Fig. 3 *C* and *D*). The steady-state kinetic parameters of the isethionate cleavage were determined spectrophotometrically using a coupled enzyme assay with yeast alcohol dehydrogenase (Fig. 3*E*). The apparent *k*_cat_/*K*_m_ (1.8 × 10^3^ M^−1^ s^−1^) under these conditions was lower than the catalytic efficiencies reported for other GREs (31, 32), but we did not detect turnover with any alternate organosulfonate (*SI Appendix*, Fig. S11) or with (*S*)-1,2-propanediol or choline as a substrate (*SI Appendix*, Fig. S12), strongly suggesting that isethionate is the native substrate. Characterization of the recombinant and reconstituted activase IslB and the GRE IslA of *D. desulfuricans* DSM642 yielded similar results, including selectivity for the cleavage of isethionate to sulfite and acetaldehyde, but a modest apparent *k*_cat_/*K*_m_ (1.2 × 10^3^ M^−1^ s^−1^) (*SI Appendix*, Fig. S13).

Finally, we examined the last putative enzyme from the *B. wadsworthia* taurine utilization pathway, AdhE. We found that the recombinant enzyme converted acetaldehyde to acetyl-CoA only in the presence of CoASH and NAD^+^ (*SI Appendix*, Fig. S14); it preferred NAD^+^ over NADP^+^. When we combined isethionate as substrate with activated GRE, NAD^+^, CoASH, and AdhE, we observed formation of acetyl-CoA, as confirmed by LC-MS (*SI Appendix*, Fig. S14*C*). Hence, the work with recombinant enzymes confirmed all four reactions of the taurine pathway of *B. wadsworthia* 3.1.6 (Fig. 1*B*).

## Discussion

Our efforts to decipher the molecular basis for the conversion of taurine to sulfite in strictly anaerobic gut bacteria has uncovered a previously uncharacterized GRE, isethionate sulfite-lyase (IslA) and its cognate activating enzyme (IslB). This enzyme catalyzes a radical-based desulfonation, generating acetaldehyde, which can be further metabolized to acetyl-CoA, and sulfite, which can serve as a terminal electron acceptor. The reaction mechanism for isethionate desulfonation by the GRE IslA (Fig. 3*A*) may proceed in analogy to the mechanisms proposed for 1,2-propanediol dehydratase (PD) and CutC, which have been supported experimentally (33, 34). This proposed mechanism would involve an initial hydrogen atom abstraction to form an α-hydroxyalkyl radical at C_2_ of isethionate, followed by a spin-center shift to eliminate the C_1_ sulfonate group (*SI Appendix*, Fig. S1*B*). As this type of C-S bond-cleavage reaction was not previously known to be catalyzed by GREs, the discovery of IslA further diversifies the known repertoire of radical reactions catalyzed by this superfamily (29) and adds to the limited number of characterized microbial desulfonative enzymes (17, 35), including Xsc (EC 2.3.3.15) (Fig. 1*A*), cysteate (3-sulfoalanine) sulfite-lyase (CuyA) (EC 4.4.1.25), 3-sulfolactate sulfite-lyase (SuyAB) (EC 4.4.1.24), and the range of mono-oxygenases and dioxygenases (EC 1.14.-) desulfonating aromatic and aliphatic sulfonates in aerobic bacteria (36).

Remarkably, the logic of the two catabolic pathways that ultimately cleave taurine to acetyl-CoA and sulfite (Fig. 1*B*), either in the absence of molecular oxygen in strictly anaerobic bacteria by the highly oxygen-sensitive GRE IslA or in aerobic bacteria via the oxygen-insensitive TDP-dependent Xsc, parallels the catabolism of pyruvate to acetyl-CoA in *E. coli*. During anaerobic fermentation, *E. coli* can use the GRE pyruvate formate-lyase (37), while during aerobic respiration, this enzyme is replaced by a TDP-dependent, oxygen-insensitive pyruvate dehydrogenase. Furthermore, the observation that anaerobic *D. thiosulfatigenes* GKNTAU, which is highly specialized for taurine fermentation (19), uses an Xsc enzyme (18), while *B. wadsworthia* uses IslA for the respiration of the sulfite from taurine with external electrons such as from lactate or H_2_ (38), may point to an interesting adaptation for maintaining redox balance under these different anaerobic growth conditions.

**Fig. 4.**
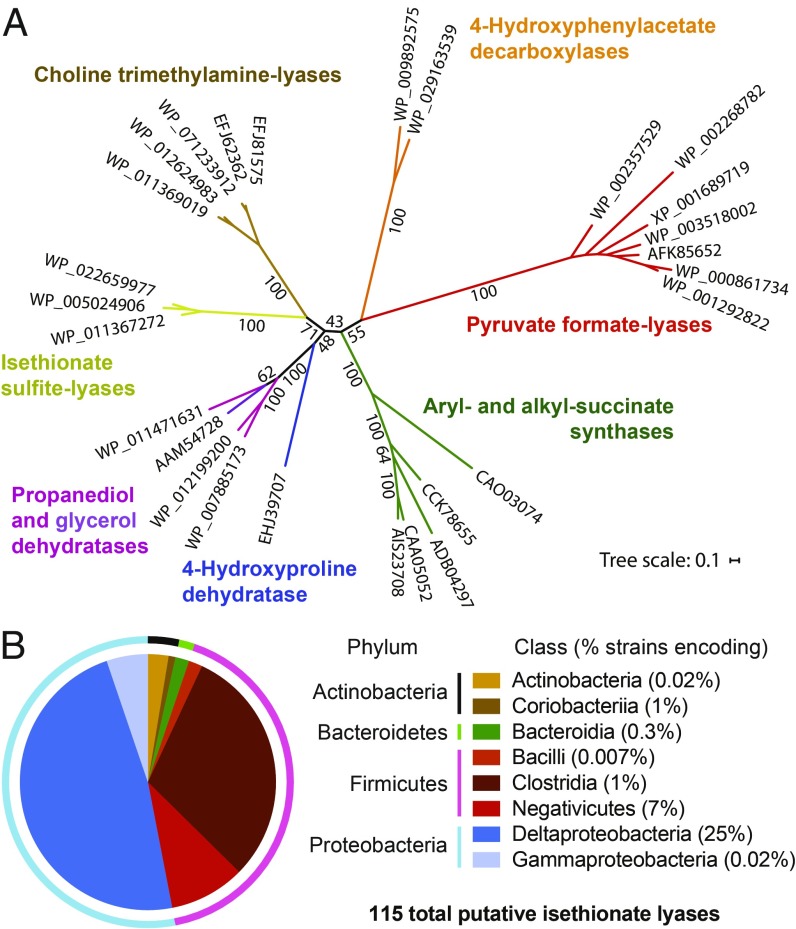
Anaerobic organosulfonate metabolism has an unexpectedly broad distribution in sequenced bacteria. (*A*) A maximum likelihood phylogenetic tree illustrating that the IslAs are phylogenetically distinct from previously characterized GREs. The listed sequence identifiers are GenBank accession codes. (*B*) Illustration of the distribution of 115 putative IslAs, as retrieved from the National Center for Biotechnology Information database at a threshold of 62% identity across sequenced bacterial genomes (*SI Appendix*, Table S1). The pie chart showing their relative taxonomic distribution on the phylum level (outer ring) demonstrates that the majority are found in genomes of Proteobacteria and Firmicutes, and at the class level (inner circle), that they are distributed predominantly across Deltaproteobacteria, Clostridia, and Negativicute genomes. The percentages shown in brackets indicate (at the class level) the number of genomes with putative IslAs relative to all known genome sequences for this class, confirming the enrichment of IslAs in the Deltaproteobacteria (encoded in 25% of all known genomes).

Searches for additional putative IslA enzymes in sequenced microbial genomes revealed more than 250 sequences in the National Center for Biotechnology Information’s nonredundant protein database (>35% amino acid identity to the *B. wadsworthia* sequence). A total of 115 of these sequences clustered together with the *B. wadsworthia* and the *Desulfovibrio* IslA sequences in a sequence similarity network at a threshold of 62% (*SI Appendix*, Fig. S15*A*) (32) and thus are likely to process isethionate or similar organosulfonate substrates. Bioinformatic analyses revealed distinctions between these sequences and GREs of known activity. For example, comparison of the sequences of characterized and putative IslAs with the sequences of other characterized GREs revealed a unique conserved glutamine residue (Q193) positioned to interact with the sulfite-leaving group in a homology model (29) (*SI Appendix*, Fig. S15 *B* and *C*). Furthermore, phylogenetic analysis (Fig. 4*A*) reveals IslA to be part of a deeply branching group of sequences previously designated as “GREs of unknown function” that typically are not colocalized with genes encoding bacterial microcompartment structural proteins (39). We note that while IslA homologs are found predominantly in Deltaproteobacteria, these enzymes are also encoded in the genomes of many Firmicutes (Clostridia, Negativicutes), as well as in several Gammaproteobacteria and Bacteroidetes (Fig. 4*B* and *SI Appendix*, Table S1). This observation suggests that anaerobic organosulfonate catabolism may be more widely distributed across microbial diversity than previously appreciated, and evaluating these organisms’ ability to metabolize isethionate, taurine, or other organosulfonates are important topics for future research.

Finally, the discovery of this gut microbial metabolic process reshapes our view of organosulfonate utilization in the human gut microbiota and unlocks new opportunities for investigating its contribution to human disease. Microbes derive several benefits from respiring organosulfonates, including the ability to occupy distinct metabolic niches. Compared with sulfate reduction, ATP expenditure is not required for generating sulfite as an electron acceptor (Fig. 1*B*). Notably, the two *Desulfovibrio* strains down-regulate APS reductase during isethionate utilization (Fig. 2*B*), while *B. wadsworthia* is unable (or has lost the ability) to use sulfate (10). Furthermore, many sulfite-respiring bacteria exhibit a hydrogenotrophic lifestyle in the human gut (11, 23, 38), and in this respect, the taurine/isethionate pathway can serve both dissimilatory and assimilatory functions when the acetyl-CoA produced from isethionate is funneled directly into gluconeogenesis (Fig. 1*B*).

The widespread distribution of these anaerobic taurine and isethionate pathways in microbiota-relevant sulfidogenic bacteria indicates an important role for organosulfonate respiration in this microbial habitat, potentially in the context of competition with sulfate-reducing bacteria and methanogenic archaea for the H_2_ produced by gut bacterial fermentation (23), or in the context of high availability of these organosulfonate substrates during consumption of high-fat (high taurocholate) (15) and/or high-meat (high taurine) diets (40). Furthermore, given that taurine is present in high concentrations in mammalian tissues (2–20 mM) (41), this pathway also may be important for *B. wadsworthia* pathogenesis. The discovery of the molecular basis for taurine metabolism in this organism and other gut microbes will not only enable future efforts to understand the biological roles of this metabolic activity in the human body, but also may inform new approaches to controlling intestinal H_2_S production and *B. wadsworthia* infections.

## Materials and Methods

The materials and methods used in this work are described in detail in *SI Appendix*, *Materials and Methods*. The *Bilophila* and *Desulfovibrio* strains were grown in carbonate-buffered mineral salts medium reduced with Ti(III)-nitrilotriacetate. Cell-free extracts were prepared by French press disruption followed by centrifugation to remove unbroken cells; these extracts were used for proteomics analysis and measurement of Tpa, SarD, and IslAB activity. Sulfite was detected by a colorimetric (fuchsin) assay as well as by HPLC after derivatization, and acetaldehyde was detected by HPLC after derivatization. A hydrophilic interaction liquid chromatography column and HPLC-MS system was used to detect taurine, alanine, sulfoacetaldehyde, and isethionate. His-tagged Tpa and SarD were produced using *E. coli* Rosetta 2 DE3 and the His-tagged GREs, AdhE, and DctP using *E. coli* BL21. The His-tagged GRE activating enzymes were overexpressed in *E. coli* BL21(DE3) *ΔiscR::kan.* Before induction, these cultures were rendered anoxic by sparging with argon. Cell lysis and enzyme purification were also done under anoxic conditions. The recombinant GREs were rendered anoxic after purification and activated by incubation in the presence of the GRE-activating enzyme, SAM, and acriflavine as a photosensitizer in Hepes-bicine buffer under ambient light. Kinetics and substrate ranges of the GREs were measured spectrophotometrically using a coupled assay with alcohol dehydrogenase, reducing the acetaldehyde to ethanol concomitant with NADH formation.

## Supplementary Material

Supplementary File
