# Supplementary material for: A glycyl radical enzyme enables hydrogen sulfide production by the human intestinal bacterium Bilophila wadsworthia
Source: Proc Natl Acad Sci U S A. 2019 Feb 4;116(8):3171–6. doi: 10.1073/pnas.1815661116 (PMC6386719; doi:10.1073/pnas.1815661116)
Supplement: Supplementary File [file pnas.1815661116.sapp.pdf]

PNAS
www.pnas.org

Supplementary Information for

A glycyl radical enzyme enables hydrogen sulfide production by the human intestinal bacterium *Bilophila wadsworthia*

Spencer C. Peck, Karin Denger, Anna Burrichter, Stephania M. Irwin
Emily P. Balskus*, and David Schleheck*

*Correspondence: balskus@chemistry.harvard.edu and david.schleheck@uni-konstanz.de

This PDF file includes:

Supplementary text (Materials and Methods)
SI References
Figs. S1 to S15
Tables S1 and S2

www.pnas.org/cgi/doi/10.1073/pnas.1815661116

Material and methods

Chemicals

Commercial chemicals were of the highest purity available and purchased from Sigma-Aldrich, Fluka, Roth, Merck or Biomol. Sulfopyruvate and sulfoacetaldehyde (as the bisulfite adduct) was synthesized as described previously (19). Racemic 3-sulfolactate and 2,3-dihydroxypropanesulfonate (DHPS) were synthesized by Thomas Huhn (Department of Chemistry, University of Konstanz) as described elsewhere (42, 43). (*S*)-1,2-propanediol was prepared as previously described (44). Luria-Bertani (LB) medium was prepared from its basic components (10 g/L tryptone, 5 g/L yeast extract, 5 g/L NaCl) or obtained from EMD Millipore or Alfa Aesar. Isopropyl β-*D*-1-thiogalactopyranoside (IPTG) was purchased from Teknova (Hollister, CA). Acetonitrile for LC-MS analyses was purchased as LC-MS grade solvent from Honeywell Burdick & Jackson or Sigma-Aldrich.

Bacterial strains

Bilophila wadsworthia 3.1.6 was kindly provided by Dr. Emma Allen-Vercoe, University of Guelph, Canada, and *Desulfovibrio desulfuricans* subsp. *desulfuricans* type-strain (DSM 642) and *Desulfovibrio alaskensis* G20 (DSM 17464) were purchased from the Leibniz Institute DSMZ-German Collection of Microorganisms and Cell Cultures, Braunschweig, Germany. The *D. alaskensis* G20 mutants (and parental strain) were kindly provided by Adam Deutschbauer at Lawrence Berkeley National Laboratory.

Culture media for *Bilophila wadsworthia* and the *Desulfovibrio* strains

Liquid cultures of *Bilophila wadsworthia* 3.1.6, *D. desulfuricans* DSM642 and *D. alaskensis* G20 were grown in a hydrogen-carbonate-buffered mineral salts medium, made with freshwater basal salts (45) plus trace elements (46), selenium-tungstate (47) and vitamins (48) solutions, and 1 mM Ti(III)-nitrilotriacetate (NTA) as the reducing agent (49), in serum bottles with butyl rubber stoppers under a gas phase of 20% CO_2 and 80% N_2. For *B. wadsworthia*, the medium was supplemented with 1,4-naphthochinone (200 µg/l), and 20 mM sodium L-lactate was added as the electron and carbon source, and either 20 mM taurine or isethionate as the electron acceptor. For the *Desulfovibrio* strains, 20 mM lactate was added and either 20 mM taurine, isethionate or sodium sulfate as the terminal electron acceptor. The cultures were incubated in the dark at 37 °C (*Bilophila)* or at 30 °C (*Desulfovibrio)*, and harvested as appropriate (see below).

For the *D. alaskensis* G20 mutational analysis, the previously described lactate/sulfate medium (50) was used with the exceptions that the 8 mM $MgSO_4$ was replaced by 8 mM $MgCl_2$ and that 52 mM Na_2SO_4 was present as the sole electron acceptor; lactate/sulfite medium contained 52 mM Na_2SO_3 as the sole electron acceptor, and lactate/isethionate medium 60 mM isethionate. For growing *D. alaskensis* on plates, the previously described MOYLS4 medium was used 22 with 1 mM Na_2S x 9 H_2O as the reducing agent and 2 mM K_2HPO_4 as the phosphorus source.

Total proteomics

Anoxic extracts prepared for the enzyme assays (see below) were used, or extracts prepared under oxic conditions when the cells were disrupted in a bullet blender (Next Advance Inc.) in 50 mM Tris-HCl buffer (pH 8.0) containing Halt protease inhibitor cocktail (Thermo Fisher Scientific). Cell debris was removed by centrifugation (15,000 *g*, 15 min, 4 °C). The extracts were subjected to peptide fingerprinting-mass spectrometry (PF-MS) at the Proteomics Facility of the University of Konstanz (www.proteomics-facility.uni-konstanz.de) as described previously (25, 26, 51) with the exception that each sample was analyzed twice on a Orbitrap Fusion with EASY-nLC 1200 (Thermo Fisher Scientific), and that Tandem mass spectra were searched against an appropriate protein database (retrieved from IMG) using Mascot (Matrix Science) and Proteom Discoverer V1.3 (Thermo Fisher Scientific) with "Trypsin" enzyme cleavage, static cysteine alkylation by chloroacetamide, and variable methionine oxidation.

Enzyme assays with cell-free extracts of *Bilophila* and *Desulfovibrio*

Cultures were harvested in the late exponential growth phase at an OD_{580} of app. 0.3 – 0.4 by centrifugation of the serum bottles (2100 *g*, 30 min, 4 °C). Cells were resuspended in anoxic Tris-HCl buffer (50 mM pH 8.0) containing 5 mM $MgCl_2$ and disrupted by three passages through a cooled French pressure cell (140 MPa) (Aminco, MD, USA), which had been flushed with N_2 gas.
Taurine-pyruvate aminotransferase was assayed in 50 mM Tris-HCl buffer (pH 9.0) containing 5 mM taurine, 10 mM sodium pyruvate, and 0.1 mM pyridoxal-5-phosphate. Assays with cell free extract were performed with 200 or 400 µL of extract per mL reaction. Heat inactivation was done by heating the enzymes to 98 °C for ten minutes in a heating block. Samples for HPLC (see below) were mixed with acetonitrile (HPLC grade) in a ratio of 7:3 immediately after sampling in order to stop enzyme reaction, centrifuged to remove precipitate (16,100 *g*, 10 min, 4 °C), and stored at -20 °C until analysis.

Sulfoacetaldehyde reductase was assayed spectrophotometrically as the oxidation of NADH recorded at 365 nm for 1 min; the standard reaction mixture (1 mL) contained 50 mM Tris-HCl (pH 8.0), 5 mM $MgCl_2$, 0.4 mM NADH and 2 mM sulfoacetaldehyde-bisulfite adduct at room temperature. The reactions were started either by addition of substrate or by addition of cell extract. Sulfoacetaldehyde disappearance and isethionate formation during the reactions was confirmed by HPLC (see below) in subsamples taken from the reactions, in which the reaction was stopped by addition of 30% (v/v) acetonitrile; these subsamples were stored at -20 °C until analysis.

Isethionate sulfite-lyase was assayed discontinuously at room temperature by monitoring the formation of the two products sulfite and acetaldehyde. The reaction mixture (1 mL) contained 20 mM isethionate, 1 mM *S*-adenosylmethionine chloride (SAM), 1 mM Ti(III)-NTA, and about 0.2 – 0.9 mg total protein in 50 mM Tris-HCl (pH 8.0) containing 5 mM $MgCl_2$. First, buffer including isethionate and SAM was degassed under vacuum in glass cuvettes with rubber stoppers, and flushed with nitrogen gas, three times each. Then, Ti(III)-NTA was added, and the reaction was initiated by the addition of anoxic crude extract, each with a syringe and needle through the rubber stoppers. At appropriate time intervals, samples (100 µL) were taken by syringes for routine quantification of sulfite by two independent methods, through a colorimetric assay and by derivatization and HPLC-UV analysis (see below), and for routine quantification of acetaldehyde by derivatization and HPLC-UV analysis (see below).

Analytical chemistry for enzyme assays with *Bilophila* and *Desulfovibrio* cell extracts

For routine HPLC with ELSD detection, a Shimadzu Prominence HPLC-DAD system coupled with a ZAM3000 ELSD detector (Schambeck SFD GmbH, Germany) was used with a SeQuant ZIC-HILIC hydrophilic interaction liquid chromatography column (5µm, 200 Å, 150 mm x 2.1 mm; Merck). The HPLC conditions were: acetonitrile as eluent A, and 0.1 M ammonium acetate buffer (pH 7.0) containing 10% acetonitrile as eluent B; total flow rate 0.3 mL/min. The elution gradient was: from 90% eluent A to 75% in 20 minutes; from 75% to 65% in 10 minutes; plateau at 65% A for 10 minutes. Under these conditions isethionate eluted at 11.5 min, taurine at 19.5 min, alanine at 20.6 min, NADH at 26.6 min, and NAD^+ at 28.7 min, while sulfoacetaldehyde eluted only poorly separated in between 5-16 min retention time. Sulfoacetaldehyde (and acetaldehyde) was determined also by HPLC-UV against authentic standards after derivatization with DNPH. Samples were mixed in a 1:1 ratio with the derivatization agent (0.5 mg/mL 2,4-dinitrophenylhydrazine [DNPH] in acetonitrile with 0.1 % H_3PO_4), after which subsamples (5 µL) were analyzed by HPLC-UV. A mixed mode C_{18} column (Luna Omega, 5 µm, PS,

C18, 100 Å, 140 mm x 3 mm; Phenomenex) was used. The flow rate was 1 mL/min with acetonitrile as eluent A and 0.1 % formic acid in MilliQ water as eluent B. HPLC conditions were as follows:
Three minutes at 25 % A, gradient from 25 to 80 % A for ten minutes, reequilibration to 25 % A for seven minutes. Under these conditions, DNPH-derivatized sulfoacetaldehyde eluted at 5.7 min, derivatized acetaldehyde at 6.8 min, and free DNPH at 8.8 min.
Sulfite concentration in enzyme reactions was routinely determined by a colorimetric assay described previously (19). Samples taken from the reactions were immediately added in a ratio of 1:20 to a mixture of 0.56 M H_2SO_4, 0.16 ‰ (w/v) fuchsine and 0.16 % formaldehyde. The absorption at 580 nm was measured after 10 minutes against sulfite standards prepared from a fresh sodium sulfite stock solution.
Sulfite in enzyme reactions was also determined by HPLC-UV after derivatization with *N*-(9-acridinyl)maleimide (see further below) against authentic standards; the column and gradient system for analysis of DNPH-derivatized aldehydes (see above) was used for separation of the sulfite reaction product, which eluted at 3.8 – 4.2 min under these conditions.

PCR primers and DNA sequencing

Primers for cloning were synthesized by Microsynth AG (Balgach, Switzerland), Integrated DNA Technologies (Coralville, IA) or Sigma Aldrich (Table S2). PCR was conducted on Biometra T-Gradient or BioRad C1000 thermocyclers. Constructed plasmids were sequenced by GATC Biotech (Konstanz, Germany) or Eton Biosciences (Charlestown, MA). DNA sequencing results were visualized in Geneious 8.1.7, Jalview 2.9.0. or DNASTAR Lasergene 7.

Experiments with mutants of *Desulfovibrio alaskensis* G20

Transposon insertion mutagenesis was previously used to inactivate *Dde_1270*, *Dde_1272*, *Dde_1273*, *Dde_1274*, and *Dde_1275* (22). The frozen strains were revived on MOYLS4 plates amended with G418 (geneticin, 400 µg/mL) at 37 ˚C, and colonies were inoculated into Hungate tubes containing 5 mL lactate/sulfate medium amended with 800 µg/mL G418. For confirmation of specific transposon insertion by PCR, 20 µL of outgrown lactate/sulfate culture was heated at 95 ˚C for 10 min, and the lysed cells were diluted 1:10 in nuclease-free H_2O and used as PCR template. The reactions (10 µL) contained 1 µL template, 0.5 µM pRL27_IE_rev1 and 0.5 µM gene-specific primer (see Table S2), and 5 µL 2x Q5 polymerase master-mix. The PCR program was: Denaturation for 12 min at 95 °C, followed by 25 cycles of 30 s denaturation at 95 °C, 30 s annealing at 55 °C, and 90 s elongation at 72 °C. Specific transposon

insertion for each mutant was confirmed by the presence of an amplicon at 800-1000 bp on agarose gels. The growth of the mutants in lactate/sulfite and lactate/isethionate medium was monitored also in 96-well plates with a plate reader in an anoxic chamber. Therefore, precultures in Hungate tubes containing 5 mL lactate/sulfate medium (wildtype) or 5 mL lactate/sulfate medium amended with 800 µg/mL G418 (mutants) were grown at 37 ˚C for 48 h, and 100 µL of each culture was inoculated into fresh 5 mL medium and incubated further for 24 h. Then, 200 µL of each culture was inoculated into a well plate (in quadruplicate) containing 200 µL lactate/sulfite or lactate/isethionate medium, such that the initial OD_{600} was 0.02 (pathlength-corrected). For 30 h, the OD_{600} was recorded every 1 h with shaking every 20 min.

Heterologous over-production and purification of taurine-pyruvate amintotransferase (Tpa) and sulfoacetaldehyde reductase (SarD)

Chromosomal DNA was isolated from *B. wadworthia* 3.1.6 using the protocol published by the DOE Joint Genome Institute (JGI), “JGI Bacterial DNA Isolation CTAB-2012” (https://jgi.doe.gov/user-program-info/pmo-overview/protocols-sample-preparation-information/). The constructs for the production of recombinant and His-tagged Tpa and SarD of *B. wadworthia* were generated when the genes were amplified by PCR using Phusion polymerase (New England BioLabs) and the primers given in Table S2; the primers introduced a NdeI and XhoI site in front of the start and after the stop codons, respectively. The following PCR conditions were used: Denaturation for 90 s at 98 °C, followed by 30 cycles of 30 s denaturation at 98 °C, 30 s annealing at 68 °C, and 60 s elongation at 72 °C; final elongation was for 5 min at 72 °C. The PCR products were purified (QIAquick MiniElute Purification Kit) and blunt-end cloned into a pJet suicide vector (CloneJet PCR cloning kit, ThermoFisher Scientific). 2 µL of the reaction were transformed into chemically competent *E. coli* NovaBlue cells, by incubating 50 µL competent cells with 10 ng plasmid for 15 minutes on ice, followed by a heat shock at 42 °C for 30 s, and recovery on ice for 1 min. Then, 200 µL of SOC medium (2 % w/v tryptone, 0.5 % w/v yeast extract, 10 mM NaCl, 2.5 mM KCl, 10 mM $MgCl_2$, 20 mM glucose) was added and the cells were incubated at 37 °C for 45 minutes before streaking them onto selective LB agar plates (100 µg/mL ampicillin). Positive clones from the agar plates were selected by colony PCR using the insert primers, cultivated overnight in LB medium, and the plasmid was extracted (Zyppy Plasmid Miniprep kit, Zymo Research). Each 0.5 µg of pJet plasmid from a positive clone was digested with 20 U NdeI and 80 U XhoI (New England BioLabs) in CutSmart buffer for 2h at 37 °C. The inserts were then purified (Gel extraction Mini Spin Column kit, Genaxxon biosciences), and ligated (overnight, 16 °C; T4 ligase, NEB)

into the expression vector pET 28a(+) (Novagen), which had been linearized by digestion with NdeI and XhoI as described above. 2 µL of the ligation reaction were transformed into chemically competent *E. coli* NovaBlue and the cells streaked onto selective LB plates (30 µg/mL kanamycin). Positive clones were selected by colony PCR, and the constructs confirmed by DNA sequencing (GATC Biotech).

For heterologous overproduction of Tpa and SarD, the pET28 constructs were transformed into chemically competent *E. coli* Rosetta 2 DE3 cells as described above, and the cells grown in 5 mL selective LB medium (30 µg/mL kanamycin, 35 µg/mL chloramphenicol) at 37 °C overnight. 100 mL LB medium with kanamycin and chloramphenicol were inoculated with 2 mL of this overnight culture and grown at 37 °C with shaking. When the cultures reached an OD_{580} of 0.5, expression was induced by addition of IPTG to a concentration of 1 mM. The cells were incubated further for 6 hours at 37 °C, and harvested by centrifugation (26,000 *g*, 15 min, 4 °C). Cells were resuspended in 2 mL 20 mM potassium phosphate buffer (pH 7.2) containing 500 mM NaCl, 2.5 mM $MgCl_2$, 10 U DNaseI, protease inhibitor (Halt, ThermoFisher Scientific) and 20 mM imidazole (buffer A). Cells were disrupted by four passages through a cooled French pressure cell (140 MPa). Whole cells and debris were removed from the extracts by centrifugation (17,000 × *g*, 15 min, 4 °C). The His-tagged proteins were purified using affinity columns (His SpinTrap kit, GE Healthcare) with washing steps at 20, 40 and 60 mM imidazole in buffer A, followed by elution with 500 mM imidazole in buffer A. The imidazole buffer was exchanged by gel filtration (Illustra™ Nap™-5 columns, GE Healthcare) against phosphate buffer (20 mM, pH 7.2) containing 5 mM NaCl, and SDS-PAGE was used to identify the fractions containing protein and its purity (see below). The recombinant and purified proteins were stored at -20 °C in glycerol (30% v/v final concentration). For purification and storage of Tpa, pyridoxal 5'-phosphate (0.1 mM) was added to all buffers.

Heterologous over-production and purification of glycyl radical enzymes (GREs), GRE activating enzymes, acetaldehyde dehydrogenase (AdhE) and substrate binding protein (DctP)

The GRE genes were assembled into pET28a plasmids, and the GRE activating enzyme, AdhE, and DctP genes were assembled into pET29b plasmids, each *via* Gibson assembly. Inserts were prepared when 20 ng of genomic DNA of *B. wadsworthia* or *D. desulfuricans* (prepared as described above) was mixed with primers (0.5 µM of each, see Table S2) and 1x Phusion master-mix (New England BioLabs) on a 50 µL scale. The inserts were amplified using the following protocol: Denaturation for 30 s at 95°C,

followed by 25 cycles of 30 s denaturation at 95 °C, 30 s annealing at 66 °C, and 90 s elongation at 72 °C. The amplifications were assessed on agarose gel, and the amplicons purified using an Illustra GFX PCR DNA and Gel Band Purification Kit (GE Healthcare). Linearized vector was prepared by incubating 5 µg of circular empty vector with 60 U each of NdeI and XhoI in 1x CutSmart buffer on a 50 µL scale at 37 ˚C for 1 h. The vector was then purified using an Illustra GFX PCR DNA and Gel Band Purification Kit. Each insert was assembled into the vector using Gibson assembly by incubating vector (50 ng, pET28a or pET29b) with the insert (3 equivalents) in 1x Gibson Assembly Master-mix (New England Biolabs) on a 10 µL scale at 50 ˚C for 1 h. Then, 2 µL from each reaction was transformed into 50 µL chemically competent *E. coli* Top10 cells by incubating them on ice for 2 min, incubating the cells and DNA at 42 ˚C for 30 s, and recovering on ice for 1 min; SOC medium (see above) (200 µL) was added and the cells were incubated at 37 ˚C for 1.5 h. The cells were plated on LB supplemented with kanamycin (50 µg/mL, hereafter referred to as LB-Kan50) and then grown at 37 ˚C overnight. Individual colonies were inoculated into 5 mL LB-Kan50 and grown overnight at 37 ˚C. The plasmids were isolated using an E.Z.N.A. Plasmid Mini Kit I (Omega Bio-tek, D6942-02), and the plasmid inserts were confirmed to have the desired sequence by sequencing (Eton Bioscience).

The expression host *E. coli* BL21(DE3) *ΔiscR* (52) for expression of the GRE activating enzyme (see below) was constructed as follows. The strain *E. coli* BL21(DE3) *ΔiscR::kan* was generated using P1 transduction with *E. coli* strain JW2515-3 (Coli Genetic Stock Center), which contains the *ΔiscR777::kan* mutation, as the donor strain and *E. coli* BL21(DE3) as the recipient strain as previously described (53). The antibiotic resistance cassette was removed from *E. coli* BL21(DE3) *ΔiscR::kan* to produce *E. coli* BL21(DE3) *ΔiscR* using the plasmid pCP20 (Coli Genetic Stock Center) (54, 55).

Recombinant enzymes that required anoxic conditions (see below) were handled in an anoxic cabinet (MBraun) (100% N_2 atmosphere) or an anoxic soft vinyl chamber (Coy Laboratories) (97% N_2/3% H_2 atmosphere). Samples were routinely rendered anoxic by one of the following methods. Consumable goods were brought into the glovebox the day before being used. Solid chemicals were brought into the anoxic chamber in Eppendorf tubes that had been perforated. Protein solutions were rendered anoxic as described in the specific section related to their purification. Media components were routinely rendered anoxic by sparging them with argon or N_2 prior to sterilization.

For heterologous overexpression, 50 ng of plasmid was transformed into 50 µL chemically competent *E. coli* BL21, or chemically competent *E. coli* BL21 Δ*iscR* (GRE activating enzymes). The cells were incubated on ice with DNA (5 min), incubated at 42 ˚C (30 s), and recovered on ice (2 min); SOC (200

μL) was added, and the cells were shaken at 37 ˚C for 1 h. Cells were then plated on LB-Kan50 and grown overnight. Single colonies were inoculated into 5 mL LB-Kan50, and these cultures transferred further into 100 mL LB-Kan50, if appropriate. For expression of GREs and the GRE activating enzymes, an entire 100 mL starter culture was inoculated into 4 L LB-Kan50, which was split equally between two 4 L shake-flasks, and for AdhE and DctP, a 5 mL starter culture was inoculated into 1 L LB-Kan50. For overexpression of the activase, the LB medium was supplemented with glucose (1% w/v) and Fe(III)-ammonium-citrate (2 mM). The cultures were grown at 37 ˚C until they reached an OD_{600} of 0.6, at which point IPTG (0.3 mM) was added. Then, the temperature was lowered to 15 ˚C and the cultures incubated overnight. At the point of induction, the cultures expressing the GRE activating enzymes were additionally sparged with argon for 20 min, and cysteine (2 mM) and sodium fumarate (20 mM) were added, before the cultures were sealed with screw-cap tops. These cultures were then transferred into the anoxic chamber that had been chilled to 15 ˚C and incubated overnight without shaking.

For the preparation of the GRE activating enzymes, all subsequent steps took place at 4 ˚C in an anoxic chamber unless otherwise specified. For the steps taking place outside of the anoxic chamber (centrifugation and incubation on a nutator), the containers were sealed with electrical tape before removing them from the chamber.

After overnight growth, the cells were harvested by centrifugation (6,770 *g*, 10 min). The supernatant was decanted, and the cells were resuspended in 35 mL lysis buffer. For the GREs, the lysis buffer was 50 mM HEPES pH 7.5, 200 mM NaCl, 20 mM imidazole, for the GRE activating enzymes the same buffer supplemented with lysozyme (8 mg), half of an EDTA-free protease inhibitor tablet (Sigma S8830) and DTT (5 mM); for AdhE and DctP, 50 mM HEPES pH 8.0, 200 mM NaCl and 20 mM imidazole was used.

For the GREs, AdhE and DctP, the cells were lysed by two passages through an Avestin EmulsiFlex-C3 cell disruptor (35-70 MPa). The lysates were clarified by centrifugation (30 min, 20,000 *g*). For the GRE activating enzymes, the cells were incubated with the lysozyme at 4 ˚C for 1 h. The cells were then lysed by sonication with a ½" horn (7 min total sonication, 10 s on, 30 s off, 25% amplitude) in a cold water bath. The dark gray solution was transferred into a fresh 50 mL plug-seal conical vial that was sealed with electrical tape, and the lysates were clarified by centrifugation (20,000 *g*, 30 min).

For protein purification, the supernatant was incubated with Ni-NTA resin (Qiagen) (6 mL for the GREs, 3 mL for the activating enzymes, 1 mL for AdhE and DctP) that had been equilibrated with 10 column volumes of the respective lysis buffer for 1 h. The resin was pelleted (500 *g*, 5 min), and the supernatant

was decanted. The resin was resuspended in a minimal volume of lysis buffer and then transferred into a column, except for purification of the GREs, where the mixture was directly decanted into a column. After the flowthrough was collected, the resin was washed with 50 mL lysis buffer (10 mL for AdhE and DctP). The proteins were eluted by sequential washes with elution buffer (50 mM HEPES pH 7.5, 200 mM NaCl, 250 mM imidazole, pH 8.0 for the acetaldehyde dehydrogenase); for the GREs, this was four steps with 6 mL each, for the activating enzymes three steps of 4 mL, and for AdhE four steps of 1 mL. SDS-PAGE was used to identify the fractions containing the proteins and their purity.

Purified proteins were loaded into a dialysis cassette of an appropriate size; for GREs, the dialysis cutoff was 20 kDa MWCO, and 10 kDa for activating enzymes, AdhE and DctP. The proteins were dialyzed three times against 1.3 L dialysis buffer (50 mM HEPES pH 7.5, 50 mM NaCl, 10% (v/v) glycerol) for two 2 h steps and one overnight step; for AdhE and DctP, one of the 2 h steps was omitted.

In order to render the GREs anoxical, the dialyzed protein solution was concentrated *via* repeated spins in a 20 mL 30 kDa centrifuge filter (3,220 *g*, 10-20 min spins) until a volume of 5 mL was reached. Thereafter, degassing was accomplished by 12 cycles of a short vacuum pull followed by refilling with argon, then a 5 min pull of vacuum and a 5 min refill with argon; the entire process was repeated two times. Then the flasks were sealed and transferred into the anoxic chamber (97% N_2/3% H_2). Finally, all proteins were aliquoted into 200 µL portions in cryovials fitted with an O-ring, flash frozen in liquid N_2, and stored at -80 ˚C. The cryovials with GREs and activating enzymes were sealed in anoxic Hungate tubes before freezing.

In vitro assays for recombinant Tpa and SarD

Enzyme assays were conducted in 50 mM Tris-HCl buffer at a pH of 9.0 containing 5 mM taurine, 5 mM sodium pyruvate, and 0.1 mM pyridoxal-5-phosphate. Typically, 50 µg/mL Tpa and 25 µg/mL SarD were used in an assay, and SarD and 2 mM NADH were added to the reactions after 90 minutes. Samples taken for HPLC were mixed with acetonitrile (HPLC grade) in a ratio of 7:3 immediately after sampling and stored at -20 °C until analysis.

For separation of taurine, isethionate, sulfoacetaldehyde, alanine, pyruvate, NAD^+, NADH, sulfate and sulfite, the HPLC DAD ELSD system (see above) fitted with the hydrophilic interaction liquid chromatography column (ZIC-HILIC, see above) was used as follows: acetonitrile as the eluent A, and 0.1 M ammonium acetate buffer (pH 7.0) containing 10% acetonitrile as the eluent B; total flow rate 0.3 mL/min. The elution gradient was: from 90% eluent A to 75% in 20 minutes; from 75% to 65% in 10

minutes; plateau at 65% A for 10 minutes. Under these conditions, pyruvate eluted at 6.6 min, isethionate at 11.5 min, alanine at 20.6 min, NADH at 26.6 min, and NAD^{+} at 28.7 min. Sulfoacetaldehyde eluted only poorly separated in between 5-16 min retention time, and taurine eluted at 19.5 min but co-eluted with the peaks for Tris and sodium.

For HPLC with ESI-MS-MS detection, an Agilent 1100 HPLC system fitted with the same chromatography system was connected to a Thermofisher LCQ ion trap mass spectrometer. The fragmentation spectra for each analyte were recorded from the total ions (TIC) with the following ranges (exact mass of the $[M-H]^{-}$ ion as well as the mass determined in the MS tuning step in brackets): taurine (m/z = 124.01/124.20): 124-125; sulfoacetaldehyde (m/z = 122.98/123.20): 123-124; isethionate (m/z = 124.99/125.20): 125-126; alanine (m/z = 89.05 for the zwitter ion): 88-90.

For the kinetic measurement of SarD, the oxidation of NADH was measured spectrophotometrically as the decrease of absorbance recorded at 365 nm for 1 min. The reaction mixture (1 mL) contained 50 mM Tris-HCl (pH 9.5), 0.128 mg recombinant protein,1 mM NADH and 0.02 – 0.6 mM sulfoacetaldehyde-bisulfite adduct at room temperature. The reactions were started by addition of sulfoacetaldehyde. Each concentration point was measured in triplicate. The mean of the calculated specific activity was plotted against the sulfoacetaldehyde concentration and the data points were fitted to the Michaelis-Menten steady-state equation ($k_{obs} = k_{cat}* [S]/(K_m + [S])$) in Origin 8.0.

In vitro **activation of GREs and glycyl radical detection and quantification by electron paramagnetic resonance (EPR) spectroscopy**

The GRE was activated in an anoxic chamber by incubating activating enzyme (80 µM), GRE (40 µM), acriflavine (100 µM), SAM (1 mM), and bicine (50 mM pH 7.5) in reaction buffer (50 mM HEPES pH 7.5, 50 mM NaCl) at 25 ˚C for 2 h under ambient light, in a 250 µL scale for EPR spectroscopy.

The entire activation mixture was then loaded into a J. Young EPR tube (4 mm outer diameter and 8” length, from Wilmad Lab-Glass), sealed, removed from the anoxic chamber, and slowly frozen in liquid N_2. Perpendicular mode X-band EPR spectra were recorded on a Bruker ElexSysE500 EPR instrument equipped with a quartz finger dewar (Wilmad Lab-Glass) for acquiring spectra at 77 K with liquid N_2. The samples were acquired with the following parameters: microwave frequency: 9.45 GHz; power: 20 µW (40 dB attenuation); center field: 3350 Gauss; sweep width: 200 Gauss; conversion time: 20.48 ms; modulation gain: 60 dB modulation gain for samples; 30 dB for external standards; time constant: 20.48 ms; modulation amplitude: 4 G; modulation frequency: 100 kHz. Normalization due to differences in

modulation gain were automatically performed by the spectrometer. Typically, only a single scan was recorded to minimize any disruption due to bubbling from the liquid N_2. The field was calibrated by using an external standard of bisdiphenylene-β-phenylallyl (BDPA) with g = 2.0026. An external standard of Frémy salt was prepared by dissolving $K_2(SO_3)_2NO$ in anoxic 0.5 M $KHCO_3$ and diluting the standard to ~0.5 mM. The concentration of the standard was more precisely ascertained by measuring its absorbance at 248 nm (ε = 1,690 M^{-1} cm^{-1}) using a NanoDrop 2000 UV-Vis Spectrophotometer. The double integral of the Frémy salt standard was calculated on the EPR spectrometer and then used to determine the concentrations of each of the protein samples. The EPR spectra from the activation mixtures were simulated using EasySpin in MatLab using the genetic algorithm followed by the Levenberg/Marquardt algorithm after sufficient optimization. These simulations yielded the g-value, the hyperfine coupling constant, and the linewidth associated with the GRE signal.

Enzyme assays with activated GREs

The GRE was first activated as described above for EPR spectroscopy. Activated GRE (1 µM) was then added to reaction buffer (50 mM HEPES pH 7.5, 50 mM NaCl) in a 50 µl, 250 µl or 500 µL scale, as specified in the following sections, and the reaction initiated by addition of isethionate. If appropriate, reaction buffer containing yeast alcohol dehydrogenase (8 µM) and NADH (3 mM) was used, and then activated GRE and isethionate was added. In the coupled assay with yeast alcohol dehydrogenase, acetaldehyde is reduced to ethanol, which allows for monitoring the GRE reactions spectrophotometrically *via* the NADH conversion (see below). Further, without including these components, sulfite and acetaldehyde condense to form 1-hydroxyethanesulfonate, which can be indistinguishable from isethionate by LC-MS. Aerobic control reactions were first removed from the anoxic chamber and gently aerated by pipetting, prior to mixing with the isethionate.

For monitoring isethionate consumption by HPLC-MS in reactions mixtures in a 500 µL scale with 2 mM isethionate, and in the presence of yeast alcohol dehydrogenase and NADH (see above), 50 µL aliquots were withdrawn at intervals and quenched with 50 µL acetonitrile in the anoxic chamber. The samples were centrifuged (16,100 *g*, 10 min, 4 ˚C) and the supernatant was diluted into 90% acetonitrile and then analyzed under chromatographic conditions as described in the following: A ZIC-HILIC column (100 x 2.1 mm, 3.5 µm, 200 Å) was used; eluent A was 10 mM ammonium acetate in H_2O and eluent B was acetonitrile; flow rate was 0.4 mL/min; the gradient was started at 90% B, decreased to 65% B over 5 min, ramped down to 35% B over 1 min, held at 35% B for 2 min, increased to 90% B over 2 min, and

reequilibrated at 90% B for 4 min. Single ion monitoring was used to record the abundance of the $[M-H]^-$ charge state of isethionate (125.1 *m*/*z*, 0.5 *m*/*z* span, 50 ms dwell); the capillary temperature was 275 ˚C, the capillary voltage was 140 V, the source voltage offset was 20 V, the source voltage span was 20 V, the source gas temperature was 200 ˚C, and the ESI voltage was 2500 V.

For sulfite quantification and testing of the substrate range, reactions mixtures in a 50 µL scale in the presence of yeast alcohol dehydrogenase and NADH (see above) were incubated with 10 mM isethionate, ethanesulfonate, sulfoacetate, 3-sulfolactate, sulfopyruvate, taurine, hypotaurine or L-cysteine sulfinic acid, and transferred after 1 h out of the anoxic chamber and derivatized according to a modified procedure that was previously reported (56). A 100 mL solution of 0.3 M boric acid, 0.3 M KCl, and 0.02 M Na_2-EDTA was mixed with a 50 mL solution of 0.3 M Na_2CO_3 and 0.02 M Na_2-EDTA to adjust the solution of the mixture to pH 8.8. 150 µL of this solution was added to each reaction, followed by 50 µL of an acetone solution containing *N*-(9-acridinyl)maleimide (0.1% w/v). Freshly prepared sodium sulfite standards were derivatized at the same time. The reactions were incubated at 37 ˚C for 2 h, and then transferred to a UV-transparent 96-well plate. The fluorescence intensity was recorded using a Spectramax i3 Plate Reader. The excitation wavelength was 362 nm (9 nm bandwidth); the emission wavelength was 436 nm (15 nm bandwidth). The photomultiplier setting set to "low" and 2 flashes per read were used.

For acetaldehyde quantification, reactions in a 50µl scale with 10 mM isethionate were transferred after 1 h out of the anaerobic chamber and quenched by addition of 50 µL of derivatization solution (3.8 mg 2,4-dinitrophenylhydrazine [DNPH] dissolved in 3 mL acetonitrile and acidified with 7.5 µL 85% H_3PO_4). Acetaldehyde standards in reaction buffer were derivatized at the same time. The samples were incubated in the dark for 1 h at room temperature, centrifuged (16,100 *g*, 10 min), and then held at 10 ˚C until analysis was carried out (<24 h). HPLC analysis was used to detect the DNPH-derivatized acetaldehyde, when separated using a Dikma C_{18} Inspire (50 x 4.6 mm, 5 µm) column. Eluent A was 0.1% formic acid in H_2O and eluent B 100% acetonitrile, and the flow rate was 1.0 mL/min. The gradient was started at 20% B, was held at 20% B for 1 min, ramped to 80% B over 1.5 min, held at 80% B for 0.5 min, decreased to 20% B over 1 min, and reequilibrated at 20% B for 2 min. The absorption at 360 nm was monitored. 5 µL of sample was injected.

For the spectrophotometrically coupled GRE assay for kinetics determination, activated GRE (0.1 µM) was mixed with yeast alcohol dehydrogenase (2 µM) and NADH (200 µM) on a 200 µL scale in a 96 well plate. The reactions were initiated by addition of isethionate (1, 2, 5, 10, 20, 30, 40 or 50 mM), and the

plate was loaded into a plate reader set to 27 ˚C. These reactions were repeated in quadruplicate. The pathlength-corrected absorbance at 340 nm was recorded every 10 s for up to 5 min. The rate of NADH disappearance was assumed to correlate 1:1 with acetaldehyde production. The initial rate of product formation was then normalized for the amount of active enzyme in solution. The observed rate constant was fit to the standard Michaelis-Menten steady-state equation ($k_{obs} = k_{cat}* [S]/(K_m + [S])$) in Graphpad Prism.

For the coupled GRE assay with the potential substrates 2-mercaptoethanesulfonate (coenzyme M), 2-hydroxyethylphosphonate (2-HEP), 2,3-dihydroxypropanesulfonate (DHPS), 3-sulfolactate, choline, and (*S*)-1,2-propanediol the GRE was first activated as described above. Then, on a 200 µL scale, activated GRE (0.1 µM for choline or (*S*)-1,2-propanediol and 0.5 µM for all other potential substrates) was mixed with NADH (200 µM) and an alcohol dehydrogenase (2 µM) that would reduce the predicted organic product: This was yeast alcohol dehydrogenase for coenzyme M, 2-HEP, choline, and (*S*)-1,2-propanediol (the predicted organic product would be acetaldehyde for all except (*S*)-1,2-propanediol which would yield propionaldehyde), glycerol dehydrogenase from *Cellulomonas* sp. for DHPS (the predicted organic product would be hydroxyacetone, which glycerol dehydrogenase has activity towards) and L-lactate dehydrogenase from rabbit muscle for 3-sulfolactate (the predicted organic product would be pyruvate). The reactions were initiated by addition of the substrates, and the plate was loaded into a plate reader set to 30 ˚C (for choline and (*S*)-1,2-propanediol) or 22 ˚C (all other substrate analogs). The pathlength-corrected absorbance at 340 nm was recorded every 10 s for up to 5 min.

Characterization of the recombinant acetaldehyde dehydrogenase AdhE

Recombinant and purified AdhE (10 µM) was incubated with CoASH (500 µM), NAD^+ or $NADP^+$ (1 mM), and acetaldehyde (10 mM) in reaction buffer (50 mM HEPES pH 8.0, 50 mM NaCl) on a 200 µL scale at 22 ˚C, with addition of the acetaldehyde last to initiate the reaction. The pathlength-corrected absorption at 340 nm was recorded every 30 s for 15 min. No reaction was observed when the reaction was run at pH 7.5; presumably the catalytic active site cysteine is protonated at this lower pH and, hence, AdhE is unable to initiate attack on the aldehyde. Addition of unactivated GRE did not stimulate formation of NADH. The reactions were quenched by addition of acetonitrile (100 µL) to each reaction. 200 µL of each sample was transferred to an Eppendorf tube, and the precipitated proteins were pelleted by centrifugation (16,100 *g*, 10 min, 4 ˚C). 100 µL of each tube was then transferred into an LC-MS vial.

For detection of CoASH and acetyl-CoA, 5 µL of each sample was injected onto the Sequant ZIC-HILIC column (see above). Eluent A was 20 mM ammonium acetate pH 8.0, and eluent B was acetonitrile. The flow rate for the column was 0.4 mL/min, and the column was started at 100% B which was decreased linearly to 0% B over 10 min, held at 0% B for 2 min, increased to 100% B over 2 min, and held at 100% B for 6 min. The absorbance at 260 nm was recorded with a 4 nm bandwidth. For MS analysis, single ion monitoring of the $[M-2H]^{2-}$ charge state for acetyl CoA (403.6 *m/z*) and CoASH (382.5 *m/z*) were recorded with a 50 ms dwell time and a 0.5 *m/z* span. The MS settings were: capillary temperature, 275 ˚C; capillary voltage, 180 V; voltage offset, 20 V; voltage span, 0 V; source gas temperature, 150 ˚C; ESI voltage, 2500 V.

Determination of DctP ligand specificity and binding constant

For determining the ligand specificity of *D. desulfuricans* DSM642 DctP, the protein (10 µM) was incubated with or without ligand (10 mM) and 5x SYPRO Orange in 50 mM HEPES pH 7.5, 50 mM NaCl on a 20 µL scale (in quadruplicates) in a bright white 96 well real-time PCR plate. The plate was loaded into a BioRad CFX 96 RT-PCR instrument. The plate was heated at 25 ˚C for 1 min, and the fluorescence was recorded. The temperature was increased by 1 ˚C per minute with a fluorescence reading recorded at the end of each minute until 95 ˚C had been reached. The fluorescence setting used was the "FRET" setting with an opaque plate setting. Ligands tested included isethionate, hypotaurine, taurine, sulfite, sulfoacetaldehyde (protected as the bisulfite adduct), sulfopyruvate, 3-sulfolactate, sulfoacetate, ethylsulfonate, vinylsulfonate, 1-hydroxyethanesulfonate, 2,3-dihydroxypropanesulfonate, 2-hydroxyethylphosphonate, and L-cysteine sulfinic acid. The melting temperature for each well was calculated by the instrument based on where the change of fluorescence in fluorescence as a function of temperature was maximal (*i.e.* d(Fluorescence)/dT was at its highest). The average melting temperature across four separate wells was subtracted from the average melting temperature across the apo wells to determine the ΔT_m. To determine the K_d of the *D. desulfuricans* DctP to isethionate, protein (10 µM) was incubated in quadruplicate with isethionate (0, 0.01, 0.025, 0.05, 0.1, 0.25, 0.5, 0.75, 1, 2, 5, 10, 20, 30 or 50 mM) and 5x SYPRO Orange in 50 mM HEPES pH 7.5, 50 mM NaCl on a 20 µL scale in a bright white 96 well rt PCR plate. The average melting temperature at each concentration was determined as above. The average ΔT_m as a function of isethionate concentration was fit in GraphPad Prism 7 to the "One site — Specific binding" equation, $\Delta T_m = \Delta T_{max} * [S] / (K_d + [S]$ (all abbreviations have the standard meaning).

Construction of multiple sequence alignments

Multiple sequence alignments were routinely constructed using the default parameters of Clustal Omega (https://www.ebi.ac.uk/Tools/msa/clustalo/) with a Pearson/FASTA output format, and the results were visualized in Geneious 8.1 (Biomatters Ltd).

Bioinformatic identification of putative sulfoacetaldehyde reductases

The distribution of the sulfoacetaldehyde reductase was examined by using the *B. wadsworthia* sequence as the query for a BLAST search in the NCBI non-redundant protein database (September 7, 2017). Authentic acetaldehyde dehydrogenases, such as CutO from the choline utilization cluster, exhibit only modest sequence identity to SarD (33% ID/E-55). To identify putative SarD homologs, we gathered only the sequences that exhibited substantially more homology than that relationship (27 sequences in total; the least similar sequence was 39% ID/5E-90 to the query sequence). The primary sequences of these proteins and several experimentally verified alcohol dehydrogenases were aligned as outlined above.

The putative metal-binding residues within all proteins (36 proteins in total, 9 of verified function including *B. wadsworthia* SarD, and 27 of completely uncharacterized function) are completely conserved (Asp193, His197, His262, and His276, numbering according to *B. wadsworthia* SarD), supporting the annotation of these sequences as group(III) alcohol dehydrogenases. Thr266 in *B. wadsworthia* SarD is aligned with His267 in FucO from *E. coli*; in the latter protein, this residue is hypothesized to be critical for interacting with, and determining the, substrate specificity of the protein (57, 58). The other 10 proteins that have a threonine at this position in the multiple sequence alignment are the most closely-related 10 proteins by sequence identity/E-score, ranging from 61%-100% ID (E-167 to under E-200). The next most similar proteins are <50% ID to the query sequence (>E-127) and contain a methionine or tyrosine at this position. By contrast, proteins other than FucO that reduce acetaldehyde or propionaldehyde also have an essential histidine at this position. Based on the residue identity of position 267 and the large discontinuity in sequence identity percentage, we believe there were only 10 other sulfoacetaldehyde reductases in the NCBI non-redundant protein database.

A homology model of the *B. wadsworthia* SarD was generated using the MPI Bioinformatics Toolkit (https://toolkit.tuebingen.mpg.de/). This protein was used as the query sequence for an HHPred search of the most recent PDB release (as of August 8, 2017), yielding a series of proteins with similarity scores of E-55 to E-61. The ten most similar, structurally-solved proteins were used as the templates for homology model construction with Modeller. The homology model, the crystal structure of 1,3-propanediol

dehydrogenase from *K. pneumoniae* (PDB ID: 3BFJ) and the crystal structure of lactaldehyde reductase from *E. coli* (PDB ID: 1RRM) were visualized in Pymol. A structural alignment in Pymol indicated that T266 in SarD occupies the same site as the catalytically essential histidine in the other two proteins, again underscoring that likely only proteins that have a Thr in this position are authentic SarD homologs.

Bioinformatic compilation of strains containing a putative isethionate sulfite-lyase

The *B. wadsworthia* IslA-GRE (GenBank accession, EFV43471) was used as the query sequence for a BLAST search of the NCBI non-redundant protein database (September 7, 2017). The 250 highest-scoring sequences were exported (down to ~35% ID/E-145 score). As many of these sequences are unlikely to be authentic isethionate sulfite-lyases, these sequences were then clustered into a sequence similarity network using Option C of the Enzyme Function Initiative's Enzyme Similarity Tool (EFI-EST; 59). The network was generated with a sequence length requirement of >750 amino acids and with an initial alignment score of E-200. The full network was downloaded, and the edge value was subsequently refined in Cytoscape 3.2 to 62% ID, as this edge value had previously been determined to be likely sufficient to separate proteins that catalyze distinct reactions into distinct sequence similarity clusters (32). The three confirmed isethionate sulfite-lyases (from *B. wadsworthia*, *D. desulfuricans*, and *D. alaskensis*) co-occurred in the same cluster with 115 GRE sequences, suggesting that this cluster encodes isethionate sulfite-lyases or other C-S bond cleaving GREs.

Multiple tactics were taken to validate this hypothesis. First, a multiple sequence alignment of these sequences was constructed as described above. A homology model was also constructed of the *B. wadsworthia* IslA by using the MPI Bioinformatics Toolkit. The protein was used as the query sequence for an HHPred search of the most recent PDB release (as of January 3, 2017), yielding structurally characterized proteins with significant sequence similarity. The top 8 returned hits had similarity scores more stringent than E-138 and represented all structurally-characterized GREs other than ribonucleotide reductase. These proteins were used as the templates for homology model construction with Modeller. The homology model, 1,2-propanediol dehydratase from *Roseburia inulinivorans* (PDB ID 1R9D), and choline trimethylamine-lyase from *D. alaskensis* (PDB ID 1FAU) were visualized in Pymol. The multiple sequence alignment and a structural alignment in Pymol indicated that residues Q193, C468, E470, and G805 in the *B. wadsworthia* IslA align with the residues critical for dehydration in 1,2-propanediol dehydratase or trimethylamine elimination in choline trimethylamine-lyase. These residues may thus dictate specificity for bisulfite elimination; notably, these four residues are universally conserved in the

115 putative isethionate sulfite-lyases. Other residues in the active site may be less than 100% conserved, and hence it will take more (structural) work to discern whether the proteins are likely isethionate sulfite-lyases or operate on other organosulfonates.

The organisms encoding these putative IslAs were tabulated. The number of sequenced strains within the NCBI non-redundant protein database in a given taxonomic class was estimated by searching the NCBI RefSeq assemblies using "Desulfovibrio[orgn] AND latest refseq[filter]", where the "Desulfovibrio" portion was replaced with the taxon of interest. Thus, we could roughly calculate the proportion of strains within a given taxonomic order that encode an isethionate sulfite-lyase.

Construction of a phylogenetic tree for the glycyl radical enzymes

A literature search was performed to identify functionally-characterized GREs. The class II choline trimethylamine-lyase proteins contain an N-terminal extension of ~300 amino acids that hinders phylogenetic tree construction. The first ~300 amino acids from these sequences were trimmed, and then the proteins (27 sequences in total) were aligned using the Clustal Omega webserver tool with the output as "Clustal w/o numbers." The file was visualized in Mega 7 and then this file was used to construct a maximum likelihood phylogenetic tree in Mega 7. A maximum likelihood Jones-Taylor-Thornton model with uniform rates for the substitution pattern with complete deletion of any gaps and 200 bootstraps was used to construct the phylogenetic tree. A total of 670 positions were used in the final analysis. The Newick tree files were uploaded to the Interactive Tree of Life webserver (https://itol.embl.de/) to annotate the trees (60).

SI References

1. Carbonero F, Benefiel AC, Alizadeh-Ghamsari AH, & Gaskins HR (2012) Microbial pathways in colonic sulfur metabolism and links with health and disease. *Front. Physiol.* 3:448.
2. Singh SB & Lin HC (2015) Hydrogen sulfide in physiology and diseases of the digestive tract. *Microorganisms* 3(4):866-889.
3. Attene-Ramos MS*, et al.* (2010) DNA damage and toxicogenomic analyses of hydrogen sulfide in human intestinal epithelial FHs 74 Int cells. *Environ. Mol. Mutagen.* 51(4):304-314.
4. Ijssennagger N, van der Meer R, & van Mil SW (2016) Sulfide as a mucus barrier-breaker in inflammatory bowel disease? *Trends. Mol. Med.* 22(3):190-199.
5. Shatalin K, Shatalina E, Mironov A, & Nudler E (2011) H_2S: a universal defense against antibiotics in bacteria. *Science* 334(6058):986-990.
6. Tomasova L, Konopelski P, & Ufnal M (2016) Gut bacteria and hydrogen sulfide: the new old players in circulatory system homeostasis. *Molecules* 21(11):e1158.
7. Wallace JL, Motta P, & Buret AG (2018) Hydrogen sulfide: an agent of stability at the microbiome-mucosa interface. *Gastrointestinal and Liver Physiology* 314:G143-G149.
8. Zhang X*, et al.* (2018) Metaproteomics reveals associations between microbiome and intestinal extracellular vesicle proteins in pediatric inflammatory bowel disease. *Nature Communications* 9:2873.
9. Baron EJ*, et al.* (1989) *Bilophila wadsworthia*, *gen. nov. and sp. nov*., a unique Gram-negative anaerobic rod recovered from appendicitis specimens and human faeces. *J. Gen. Microbiol.* 135:3405-3411.
10. Laue H, Denger K, & Cook AM (1997) Taurine reduction in anaerobic respiration of *Bilophila wadsworthia* RZATAU. *Appl. Environ. Microbiol.* 63:2016-2021.
11. Nava GM, Carbonero F, Croix JA, Greenberg E, & Gaskins HR (2012) Abundance and diversity of mucosa-associated hydrogenotrophic microbes in the healthy human colon. *ISME J.* 6(1):57-70.
12. Baron EJ*, et al.* (1992) *Bilophila wadsworthia* isolates from clinical specimens. *J. Clin. Microbiol.* 30(7):1882-1884.
13. Yazici C*, et al.* (2017) Race-dependent association of sulfidogenic bacteria with colorectal cancer. *Gut* 66(11):1983-1994.
14. Feng Z*, et al.* (2017) A human stool-derived *Bilophila wadsworthia* strain caused systemic inflammation in specific-pathogen-free mice. *Gut Pathog.* 9:59.
15. Devkota S*, et al.* (2012) Dietary-fat-induced taurocholic acid promotes pathobiont expansion and colitis in Il10-/- mice. *Nature* 487(7405):104-108.
16. Lie TJ, Pitta T, Leadbetter ER, Godchaux W, 3rd, & Leadbetter JR (1996) Sulfonates: novel electron acceptors in anaerobic respiration. *Arch. Microbiol.* 166(3):204-210.
17. Cook AM & Denger K (2002) Dissimilation of the C_2 sulfonates. *Arch. Microbiol.* 179(1):1-6.
18. Ruff J, Denger K, & Cook AM (2003) Sulphoacetaldehyde acetyltransferase yields acetyl phosphate: purification from *Alcaligenes defragrans* and gene clusters in taurine degradation. *Biochem. J.* 369(Pt 2):275-285.
19. Denger K, Ruff J, Rein U, & Cook AM (2001) Sulphoacetaldehyde sulpho-lyase (EC 4.4.1.12) from *Desulfonispora thiosulfatigenes*: purification, properties and primary sequence. *Biochem. J.* 357(2):581-586.

20. Laue H (2000) Biochemical and molecular characterization of taurine metabolism in the anaerobic bacterium *Bilophila wadsworthia*. Doctoral dissertation (University of Konstanz, Konstanz, Germany).

21. HMP-Consortium (2012) A framework for human microbiome research. *Nature* 486:215–221.

22. Kuehl JV*, et al.* (2014) Functional genomics with a comprehensive library of transposon mutants for the sulfate-reducing bacterium *Desulfovibrio alaskensis* G20. *mBio* 5(3):e01041-01014.

23. Rey FE*, et al.* (2013) Metabolic niche of a prominent sulfate-reducing human gut bacterium. *Proc. Natl. Acad. Sci. USA* 110(33):13582-13587.

24. Goldstein EJ, Citron DM, Peraino VA, & Cross SA (2003) *Desulfovibrio desulfuricans* bacteremia and review of human *Desulfovibrio* infections. *J. Clin. Microbiol.* 41(6):2752-2754.

25. Denger K*, et al.* (2014) Sulphoglycolysis in *Escherichia coli* K-12 closes a gap in the biogeochemical sulphur cycle. *Nature* 507(7490):114-117.

26. Felux AK, Spiteller D, Klebensberger J, & Schleheck D (2015) Entner-Doudoroff pathway for sulfoquinovose degradation in *Pseudomonas putida* SQ1. *Proc. Natl. Acad. Sci. USA* 112(31):E4298-4305.

27. Laue H & Cook AM (2000) Purification, properties and primary structure of alanine dehydrogenase involved in taurine metabolism in *Bilophila wadsworthia*. *Arch. Microbiol.* 174:162-167.

28. Laue H & Cook AM (2000) Biochemical and molecular characterization of taurine:pyruvate aminotransferase from the anaerobe *Bilophila wadsworthia*. *Eur. J. Biochem.* 267:6841-6848.

29. Backman LRF, Funk MA, Dawson CD, & Drennan CL (2017) New tricks for the glycyl radical enzyme family. *Crit. Rev. Biochem. Mol. Biol.* 52(6):674-695.

30. LaMattina JW*, et al.* (2016) 1,2-Propanediol dehydration in *Roseburia inulinivorans*: structural basis for substrate and enantiomer selectivity. *J. Biol. Chem.* 291(30):15515-15526.

31. Craciun S, Marks JA, & Balskus EP (2014) Characterization of choline trimethylamine-lyase expands the chemistry of glycyl radical enzymes. *ACS Chem. Biol.* 9(7):1408-1413.

32. Levin BJ*, et al.* (2017) A prominent glycyl radical enzyme in human gut microbiomes metabolizes trans-4-hydroxy-l-proline. *Science* 355(6325).

33. Levin BJ & Balskus EP (2018) Characterization of 1,2-propanediol dehydratase reveals distinct mechanisms for B_{12}-dependent and glycyl radical enzymes. *Biochemistry* 57:3222-3226.

34. Bodea S, Funk MA, Balskus EP, & Drennan CL (2016) Molecular basis of C-N bond cleavage by the glycyl radical enzyme choline trimethylamine-lyase. *Cell Chemical Biolology* 23(10):1206-1216.

35. Cook AM, Denger K, & Smits TH (2006) Dissimilation of C_3-sulfonates. *Arch. Microbiol.* 185(2):83-90.

36. Kertesz MA (2000) Riding the sulfur cycle--metabolism of sulfonates and sulfate esters in Gram-negative bacteria. *FEMS Microbiol. Rev.* 24(2):135-175.

37. Knappe JS, G. (1990) A radical-chemical route to acetyl-CoA: the anaerobically induced pyruvate formate-lyase system of *Escherichia coli*. *FEMS Microbiology Letters* 75(4):383-398.

38. da Silva SM, Venceslau SS, Fernandes CL, Valente FM, & Pereira IA (2008) Hydrogen as an energy source for the human pathogen *Bilophila wadsworthia*. *Antonie Van Leeuwenhoek* 93(4):381-390.

39. Zarzycki J, Erbilgin O, & Kerfeld CA (2015) Bioinformatic characterization of glycyl radical enzyme-associated bacterial microcompartments. *Appl. Environ. Microbiol.* 81(24):8315-8329.

40. David LA*, et al.* (2014) Diet rapidly and reproducibly alters the human gut microbiome. *Nature* 505(7484):559-563.

41. Jacobsen JG & Smith LH (1968) Biochemistry and physiology of taurine and taurine derivatives. *Physiol. Rev.* 48(2):424-511.

42. Roy AB, Hewlins MJE, Ellis AJ, Harwood JL, & White GF (2003) Glycolytic breakdown of sulfoquinovose in bacteria: a missing link in the sulfur cycle. *Appl. Environ. Microbiol.* 69(11):6434-6441.

43. Mayer J*, et al.* (2010) 2,3-Dihydroxypropane-1-sulfonate degraded by *Cupriavidus pinatubonensis* JMP134: purification of dihydroxypropanesulfonate 3-dehydrogenase. *Microbiology* 156(Pt 5):1556-1564.

44. Schaus SE*, et al.* (2002) Highly selective hydrolytic kinetic resolution of terminal epoxides catalyzed by chiral (salen)Co^{III} complexes. Practical synthesis of enantioenriched terminal epoxides and 1,2-diols. *J. Am. Chem. Soc.* 124:1307-1315.

45. Widdel F & Pfennig N (1981) Studies on dissimilatory sulfate-reducing bacteria that decompose fatty acids. I. Isolation of new sulfate-reducing bacteria enriched with acetate from saline environments. Description of *Desulfobacter postgatei gen. nov., sp. nov*. *Arch. Microbiol.* 129(5):395-400.

46. Widdel F, Kohring G-W, & Mayer F (1983) Studies on dissimilatory sulfate-reducing bacteria that decompose fatty acids III. Characterization of the filamentous gliding *Desulfonema limicola gen. nov. sp. nov*., and *Desulfonema magnum sp. nov*. *Arch. Microbiol.* 134(4):286-294.

47. Tschech A & Pfennig N (1984) Growth yield increase linked to caffeate reduction in *Acetobacterium woodii*. *Arch. Microbiol.* 137:163-167.

48. Pfennig N (1978) *Rhodocyclus purpureus gen. nov*. and *sp. nov*., a ring-shaped, vitamin B12-requiring member of the family *Rhodospirillaceae*. *Int. J. Syst. Evol. Microbiol.* 28(2):283-288.

49. Moench TT & Zeikus JG (1983) An improved preparation method for a titanium (III) media reductant. *J. Microbiol. Methods* 1(4):199-202.

50. Craciun S & Balskus EP (2012) Microbial conversion of choline to trimethylamine requires a glycyl radical enzyme. *Proc. Natl. Acad. Sci. USA* 109(52):21307-21312.

51. Schmidt A, Muller N, Schink B, & Schleheck D (2013) A proteomic view at the biochemistry of syntrophic butyrate oxidation in *Syntrophomonas wolfei*. *PloS one* 8(2):e56905.

52. Akhtar MK & Jones PR (2008) Deletion of *iscR* stimulates recombinant clostridial Fe-Fe hydrogenase activity and H_2-accumulation in *Escherichia coli* BL21(DE3). *Appl. Microbiol. Biotechnol.* 78(5):853-862.

53. Thomason LC, Costantino N, & Court DL (2007) *E. coli* genome manipulation by P1 transduction. *Curr. Protoc. Mol. Biol.* Chapter 1:Unit 1 17.

54. Cherepanov PP & Wackernagel W (1995) Gene disruption in *Escherichia coli*: Tc^R and Km^R cassettes with the option of Flp-catalyzed excision of the antibiotic-resistance determinant. *Gene* 158(1):9-14.

55. Datsenko KA & Wanner BL (2000) One-step inactivation of chromosomal genes in *Escherichia coli* K-12 using PCR products. *Proc. Natl. Acad. Sci. USA* 97(12):6640-6645.

56. Akasaka K, Matsuda H, Ohrui H, Meguro H, & Suzuki T (1990) Fluorometric determination of sulfite in wine by *N*-(9-acridinyl)maleimide. *Agric. Biol. Chem.* 54(2):501-504.

57. Montella C*, et al.* (2005) Crystal structure of an iron-dependent group III dehydrogenase that interconverts *L*-lactaldehyde and *L*-1,2-propanediol in *Escherichia coli*. *J. Bacteriol.* 187(14):4957-4966.

58. Obradors N, Cabiscol E, Aguilar J, & Ros J (1998) Site-directed mutagenesis studies of the metal-binding center of the iron-dependent propanediol oxidoreductase from *Escherichia coli*. *Eur. J. Biochem.* 258(1):207-213.

59. Gerlt JA, *et al.* (2015) Enzyme Function Initiative-Enzyme Similarity Tool (EFI-EST): A web tool for generating protein sequence similarity networks. *Biochim. Biophys. Acta* 1854(8):1019-1037.

60. Letunic I & Bork P (2016) Interactive tree of life (iTOL) v3: an online tool for the display and annotation of phylogenetic and other trees. *Nucleic acids research* 44(W1):W242-245.

SI Figure legends

Figure S1. Overview of glycyl radical enzyme (GRE) mechanism and of the desulfonation reaction for isethionate as catalyzed by IslA. **(A)** General mechanistic hypothesis for GRE function. The 5'deoxyadenosyl radical (5'dA•) generated by a GRE-activating, radical SAM enzyme is used to install a protein-centered, stable glycyl radical (Gly•) within the GRE. A thiyl radical (Cys-S•) is proposed to serve as the active oxidant in all GREs and would be generated transiently within the active site. S, substrate; P, product. **(B)** The proposed reaction mechanism for isethionate desulfonation in analogy to the mechanisms proposed for 1,2-propanediol dehydratase (PD) and choline trimethylamine-lyase (CutC), which have been supported experimentally (33, 34). Following initial hydrogen abstraction by the thiyl radical to form an α-hydroxyalkyl radical at C_2 of isethionate, IslA may perform a spin-centered shift to eliminate the sulfonate group at C_1.

Figure S2. Characterization of the DctP homolog of *D. desulfuricans* DSM642. **(A)** Recombinantly produced and purified DctP solute-binding protein was incubated without ligand (red) or with isethionate (orange), taurine (black), hypotaurine (green), sulfoacetaldehyde (blue), or ethylsulfonate (purple) in the presence of SYPRO Orange, and the increase in fluorescence was monitored while the protein was heated. Only isethionate significantly stabilized the protein to thermal denaturation. Data is shown as the mean of four technical replicates; error bars are omitted for clarity. **(B)** The melting temperature as a function of isethionate concentration was determined, and the curve was fit to a single-site binding equation. The K_d was 1.7 ± 0.1 mM. The melting temperature at each concentration represents the mean of four technical replicates; error bars indicate the standard deviation.

Figure S3. Experiments with cell-free extracts reveal a putative desulfonating GRE. Formation of sulfite and acetaldehyde from isethionate in strictly anoxic cell-free extracts prepared of taurine-grown *B. wadsworthia* and isethionate-grown *Desulfovibrio spp.* The specific activity of isethionate sulfite-lyase calculated from the rate of sulfite formation was 5.3 mU/mg

for *B. wadsworthia* and 6.6 and 3.8 mU/mg for the *Desulfovibrio* sp. strains DSM642 and G20, respectively. The reaction mixtures contained cell-free extract, isethionate, SAM and Ti(III)-NTA in Tris-HCl buffer, pH 8.0. Shown are results of representative enzyme reactions replicated at least three times with material from independent growth experiments.

Figure S4. Growth experiments with transposon mutants of *D. alaskensis* G20.
D. alaskensis without any mutation (wild-type) (**A**), or strains with a single disruption in the genes (Figure 1B) for a (**B**) putative regulator (IMG locus tag Dde_1270), (**C**) GRE-activating enzyme (*islB*, Dde_1272), (**D**) GRE (*islA*, Dde_1273), and (**E**) fused DctMQ transporter (Dde_1274) and (**F**) DctP solute-binding protein (Dde_1275) of a TRAP-transport system. Cultures were grown in either lactate/sulfite medium (blue) or lactate/isethionate medium (red). The data shown is the mean of four replicate growth experiments ± standard error of the mean after baseline correction.

Figure S5. SDS-PAGE of purified recombinant enzymes. Precision Plus Protein™ All Blue Standards (lane 1), *B. wadsworthia* Tpa (51 kDa, lane 2), SarD (42 kDa, lane 3), IslB (36 kDa, lane 4), IslA (96 kDa, lane 5), AdhE (52 kDa, lane 6), and *D. desulfuricans* IslB (36 kDa, lane 7) and IslA (96 kDa, lane 8).

Figure S6. Sulfoacetaldehyde reduction by SarD of *B. wadsworthia* 3.1.6.
The reactions contained 130 µg/mL recombinant SarD in 50 mM Tris-HCl buffer at pH 9.5. The sulfoacetaldehyde (SAA) concentration was varied between 0 – 0.6 mM in the presence of 1 mM NADH, and the initial rates of NADH consumption were determined spectrophotometrically as decrease of absorbance at 340 nm. The data shown represents the mean ± standard deviation of at least three technical replicates. The K_m^{app} was estimated at 0.51 mM ± 0.08 mM SAA and V_{max} at 351 ± 38 mU/mg protein (mean ± standard error of the mean).

Figure S7. Bioinformatic analysis of sulfoacetaldehyde reductase SarD. **(A)** Structural alignment of the crystal structures of DhaT (blue), FucO (magenta), and the homology model of the characterized SarD of *B. wadsworthia* (green) generated with Modeller. SarD reduces

sulfoacetaldehyde, DhaT reduces 3-hydroxypropionaldehyde, and FucO reduces lactaldehyde. FucO was crystallized in the presence of the product propanediol, which is abbreviated as "PD" in the figure. Labels are the same color as the structures. PDB IDs are 3BFJ for DhaT and 1RRM for FucO. (**B**) Multiple sequence alignment of putative sulfoacetaldehyde reductases compared to DhaT (blue), FucO (magenta), and SarD of *B. wadsworthia* (green label for the protein and black labels for the putative SarD homologs). The black asterisks indicate the metal-binding residues in DhaT and FucO; these residues are completely conserved in SarD of *Bilophila* and the putative SarDs. The catalytically essential His from DhaT and FucO is hypothesized to mediate a critical interaction with the substrate. In SarD and candidate SarD homologs, this residue is instead a Thr. Only the 10 most similar proteins contain a Thr at this position; other less similar proteins contain a variety of other residues at this position. The putative SarD sequences occur exclusively in the order *Desulfobacterales* and *Desulfovibrionales* of deltaproteobacteria. The listed numbering is according to the *B. wadsworthia* SarD.

Figure S8. Transformation of taurine *via* sulfoacetaldehyde to isethionate by recombinantly produced and purified Tpa and SarD of *B. wadsworthia* 3.1.6. LC-MS ion-extract chromatograms are shown when scanning for $[M-H]^-$ ions of taurine, alanine, sulfoacetaldehyde and isethionate. Top row: sample taken from a reaction mixture containing taurine (5 mM), pyruvate (5 mM) and pyridoxal-5-phosphate (0.1 mM) directly after addition of recombinant Tpa (50 µg/ml). Middle row: sample taken after 60 min incubation time, directly before the addition of recombinant SarD (25 µg/ml) and NADH (1 mM). Bottom row: sample taken after 20 min additional incubation time. No formation of alanine, sulfoacetaldehyde and isethionate was detectable in absence of the cosubstrates pyruvate and NADH, respectively. Note that also the sulfoacetaldehyde standard was only poorly separated by hydrophilic interaction liquid chromatography (Material and Methods).

Figure S9. Reduction of IslB of *B. wadsworthia* and *D. desulfuricans* as characterized by UV-visible spectroscopy. Recombinant and purified (**A**) *B. wadsworthia* or (**B**) *D. desulfuricans*

GRE-activating enzyme (10 μM) was incubated without sodium dithionite (red line) or with sodium dithionite (50 μM, blue line). For both enzymes, sodium dithionite abated the broad absorption feature of the oxidized iron-sulfur chromophore at 410 nm.

Figure S10. Isethionate consumption by the *B. wadsworthia* IslA. Activated IslA (1 μM) was incubated with isethionate (2 mM) and at selected timepoints, a portion of the reaction was withdrawn and quenched for determination of the residual isethionate by LC-MS. The reaction shown was conducted in the presence of yeast alcohol dehydrogenase (8 μM) and NADH (3 mM) in order to also monitor acetaldehyde formation as NADH consumption (see Material and Methods and Figs. 3D and S10CD).

Figure S11. Alternative substrates evaluated for turnover by IslA. (**A**) A range of substrates was evaluated for turnover either by quantifying sulfite formed or by monitoring the formation of the predicted carbon-containing product using a coupled enzyme assay. (**B**) For certain substrates, sulfite was quantified after incubations with the *B. wadsworthia* (red) or *D. desulfuricans* (blue) IslA, or without enzyme (gray). Data is shown as the average ± the standard deviation of three technical replicates. Activity was only observed with isethionate as substrate. (**C**) The *B. wadsworthia* (solid traces) or *D. desulfuricans* (dashed traces) IslA were incubated with isethionate (black), taurine (blue), or hypotaurine (red) in the presence of yeast alcohol dehydrogenase and NADH; formation of aldehydes was monitored by the consumption of NADH. Activity was only observed with isethionate as the substrate. (**D**) The *B. wadsworthia* (solid traces) or *D. desulfuricans* IslA (dashed traces) were incubated with isethionate (black), 2-mercaptoethanesulfonate (orange), 2-hydroxyethylphosphonate (yellow), 2,3-dihydroxypropanesulfonate (green), or 3-sulfolactate (magenta) in the presence of an appropriate alcohol dehydrogenase (see Material and Methods) and NADH. Activity was only observed with isethionate as substrate.

Figure S12. Substrates of other GREs evaluated for turnover by IslA. Choline is processed by the GRE choline trimethylamine-lyase (CutC) to generate acetaldehyde and trimethylamine; (*S*)-1,2-propanediol is turned over by the GRE (*S*)-1,2-propanediol dehydratase (PD) to yield

propionaldehyde and H_2O. Recombinant IslA from *B. wadsworthia* was incubated with 50 mM choline (cyan), 50 mM (*S*)-1,2-propanediol (red), or 50 mM isethionate as a positive control (black) in the presence of yeast alcohol dehydrogenase and NADH. Formation of aldehydes was monitored by consumption of NADH. Activity was only observed with isethionate as the substrate.

Figure S13. Characterization of the *D. desulfuricans* IslA. (**A**) A representative EPR spectrum of the recombinant and purified *D. desulfuricans* IslA is shown. Simulation of the data revealed that the radical has parameters similar to those previously reported for other GREs (g = 2.0034, A = 1.4 mT). Comparison to an external standard indicated that 38 ± 3% of all GRE polypeptides are activated (mean ± standard deviation of three replicates). (**B**) Sulfite and (**C**) acetaldehyde formation was observed only with activated IslA and isethionate as substrate under strictly anoxic reaction conditions, and no reaction was observed when oxygen was present or either the activase, GRE, or the substrate was omitted. (**D**) The Michaelis-Menten steady-state kinetics of isethionate cleavage were determined using the spectrophotometric coupled enzyme assay with yeast alcohol dehydrogenase and NADH. The k_{cat} was 12 ± 0.2 s^{-1}, and the K_m was 6.3 ± 0.5 mM. Data in this figure is shown as the mean ± standard deviation of at least three technical replicates.

Fig. S14. Characterization of the *B. wadsworthia* CoA-acylating acetaldehyde dehydrogenase (AdhE).

(**A**) A spectrophotometric assay with recombinant and purified AdhE demonstrated conversion of NAD^+ to NADH in the presence of all reaction components (red) but strongly reduced conversion with $NADP^+$ instead of NAD^+ (blue). No conversion was observed when protein (orange), CoASH (green), NAD^+ (purple), or acetaldehyde (gray) was omitted. (**B**) The reactions from panel A were analyzed by LC-MS for the conversion of CoASH (red traces) to acetyl-CoA (blue traces). Robust conversion was only observed in the full reaction with NAD^+. (**C**) Acetaldehyde was generated *in situ* by the action of the recombinant *Bilophila* sulfite-lyase when

cleaving isethionate, and the AdhE converted CoASH (red traces) to acetyl CoA (blue traces), as determined by LC-MS analysis.

Fig. S15. Bioinformatic characterization of the isethionate sulfite-lyases.
(**A**) Sequence similarity network of the 250 sequences most similar to the *B. wadsworthia* isethionate sulfite-lyase (IslA) amino acid sequence. The edge threshold was set to a minimum of 62 % identity. At this edge value, the three IslA characterized in this study occur in the same cluster. The node in red represents IslA of *B. wadsworthia*, and blue IslA of *D. desulfuricans*, orange IslA of *D. alaskensis*, and gray uncharacterized proteins. (**B**) Multiple sequence alignment of selected GREs. The starred residues denote positions in CutC and PD that are critical for catalysis; numbering is according to *B. wadsworthia* IslA. All sequences of characterized IslAs conserve Q193, C468, E470, and G805, as well as all candidate IslAs, for which three representative sequences from *Bacteriodales* bacterium (Bbac), *Bifidobacterium tissieri* (Btis) and *Clostridium butyricum* (Cbut) are shown. For a complete list of all 115 candidate IslA or of other C-S bond cleaving GREs, see Table S1. (**C**) Alignment of the crystal structures of CutC (brown), PD (magenta), and the homology model of *B. wadsworthia* IslA (yellow) generated with Modeller. In addition to the substrates of CutC and PD, choline ad (*S*)-1,2-propanediol, respectively, the residues identified from panel B are also shown; labels are the same color as for the structures. The homology model suggests that Q193 of IslA might facilitate elimination of sulfite in the same way as Asp216 does for trimethylamine in CutC and His166 does for water in PD. The importance of the residues in this position have been noted by others as predictive of the reactivity of the GRE (29). PDB IDs are 5FAU for CutC and 5I2G for PD.

Fig. S1

A

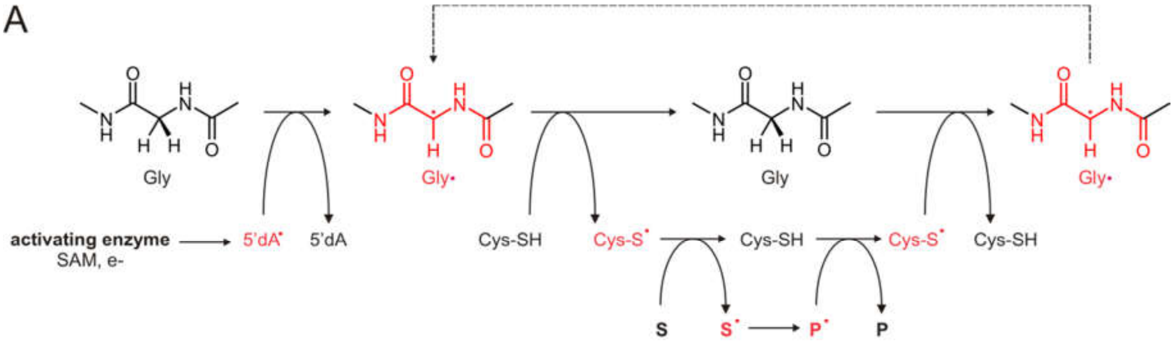
Gly
Gly•
Gly
Gly•
activating enzyme
SAM, e-
5'dA•
5'dA
Cys-SH
Cys-S•
Cys-SH
Cys-S•
Cys-SH
S
S•
P•
P

B

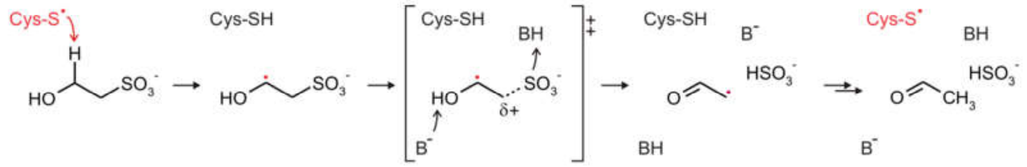
Cys-S•
Cys-SH
Cys-SH
BH
B-
Cys-SH
B-
HSO3-
BH
Cys-S•
BH
HSO3-
B-

Fig. S2

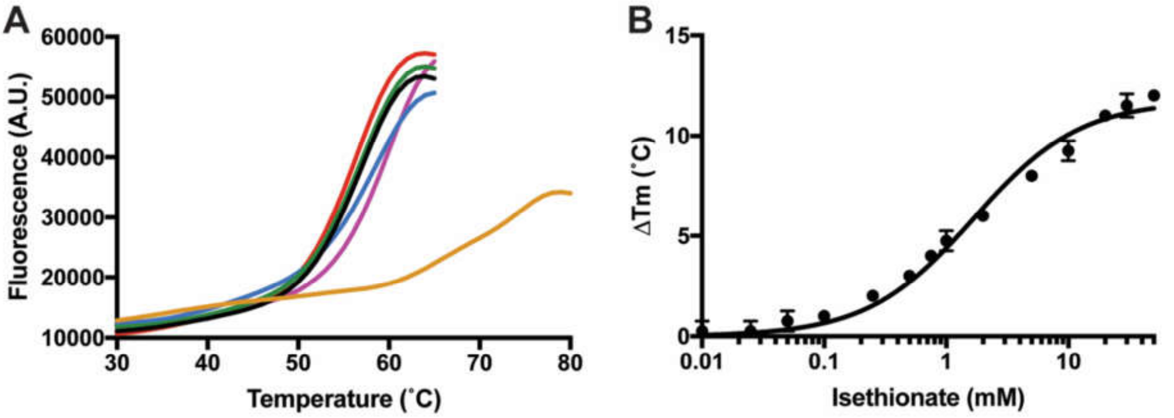

A
60000
50000
40000
30000
20000
10000
Fluorescence (A.U.)
30
40
50
60
70
80
Temperature (˚C)
B
15
10
5
0
ΔTm (˚C)
0.01
0.1
1
10
Isethionate (mM)

Fig. S3

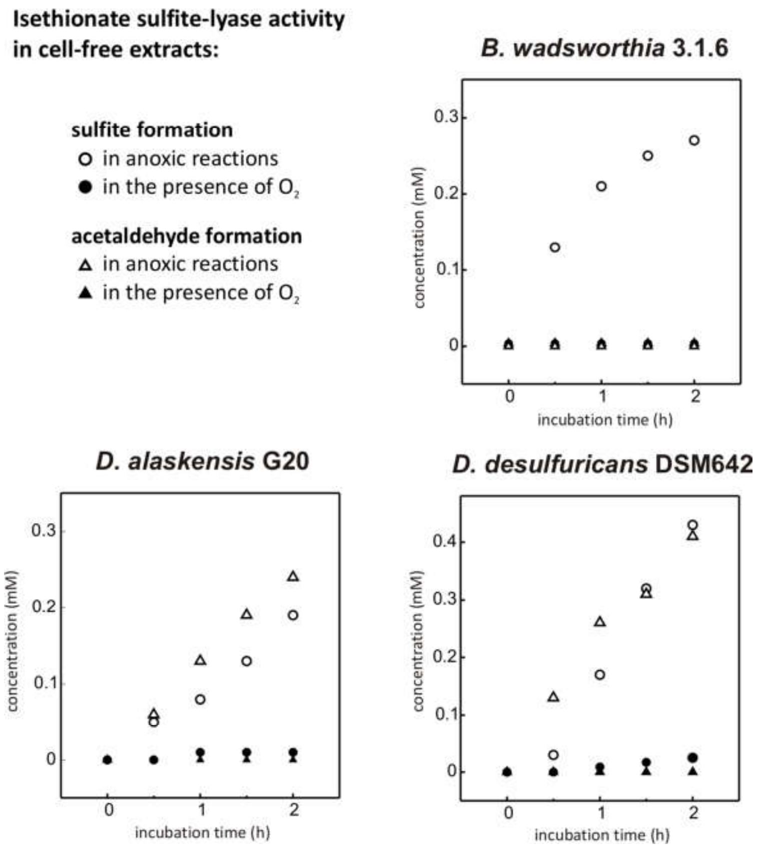

Isethionate sulfite-lyase activity
in cell-free extracts:
sulfite formation
in anoxic reactions
in the presence of O2
acetaldehyde formation
in anoxic reactions
in the presence of O2
B. wadsworthia 3.1.6
D. alaskensis G20
D. desulfuricans DSM642
concentration (mM)
incubation time (h)
0
0.1
0.2
0.3
0.4
0
1
2

Fig. S4

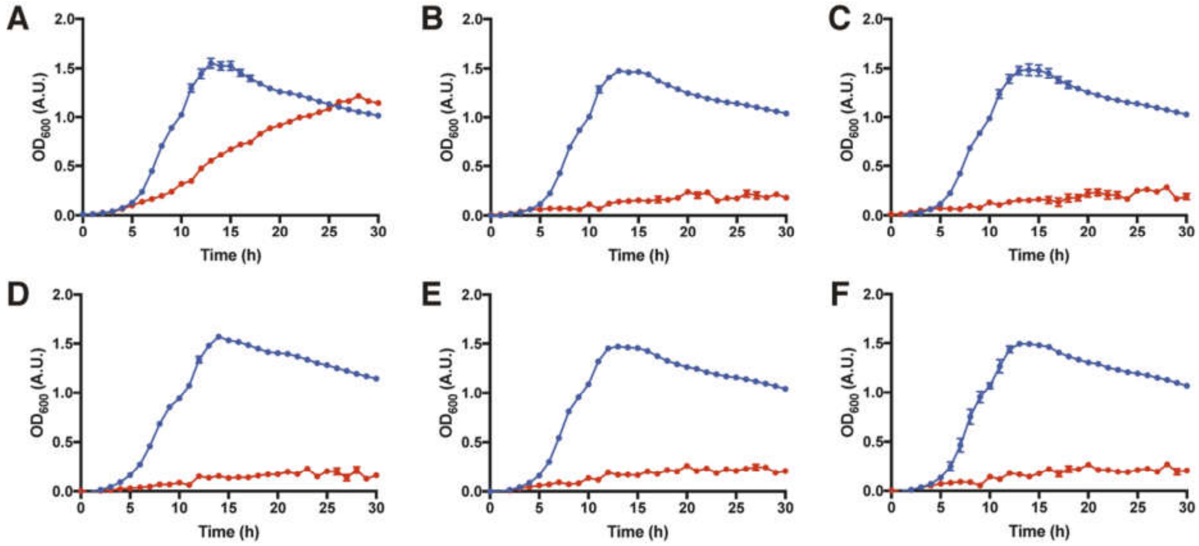

Fig. S5

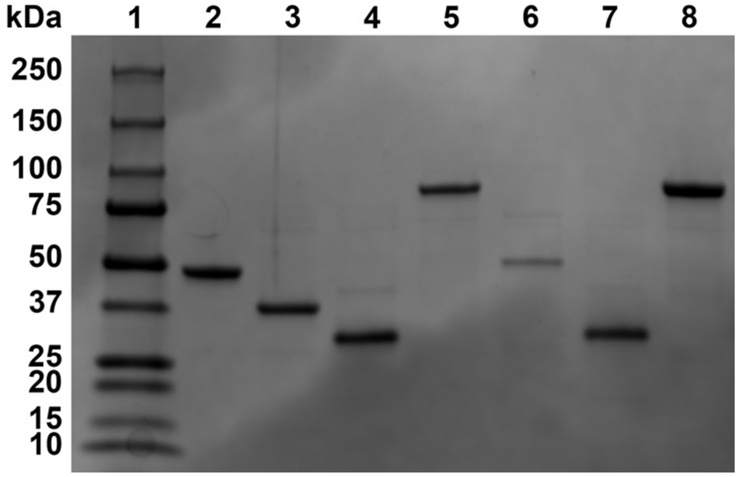

Fig. S6

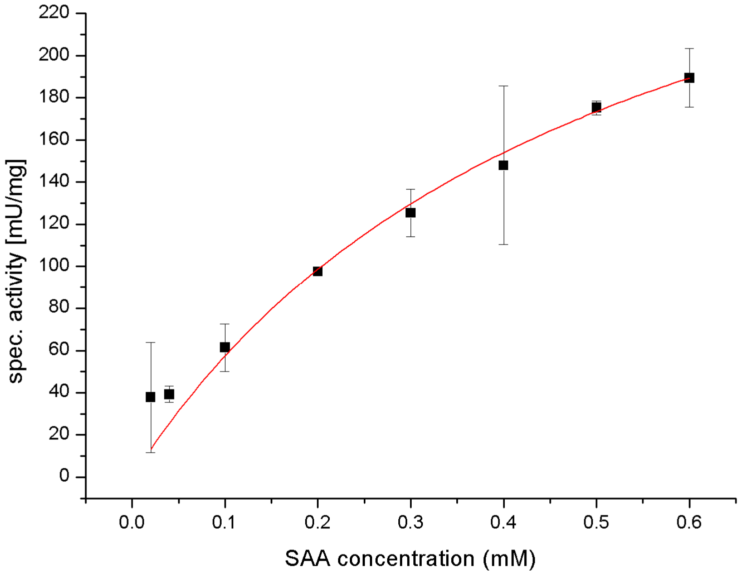

spec. activity [mU/mg]
SAA concentration (mM)
0
20
40
60
80
100
120
140
160
180
200
220
0.0
0.1
0.2
0.3
0.4
0.5
0.6

Fig. S7

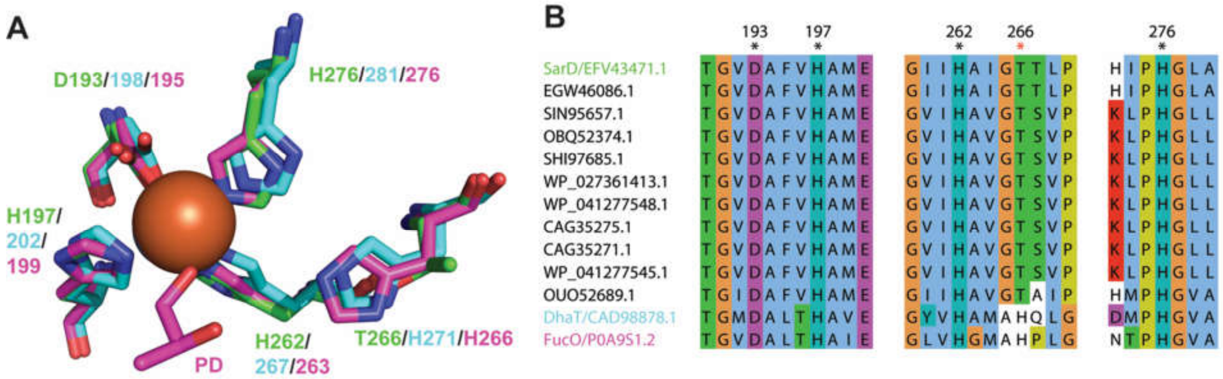
A
D193/198/195
H276/281/276
H197/
202/
199
PD
H262/
267/263
T266/H271/H266
B
193 197 262 266 276
SarD/EFV43471.1 TGVDAFVHAME GIIHAIGTTLP HIPHGLA
EGW46086.1 TGVDAFVHAME GIIHAIGTTLP HIPHGLA
SIN95657.1 TGVDAFVHAME GVIHAVGTSVP KLPHGLL
OBQ52374.1 TGVDAFVHAME GVIHAVGTSVP KLPHGLL
SHI97685.1 TGVDAFVHAME GVIHAVGTSVP KLPHGLL
WP_027361413.1 TGVDAFVHAME GVIHAVGTSVP KLPHGLL
WP_041277548.1 TGVDAFVHAME GVIHAVGTSVP KLPHGLL
CAG35275.1 TGVDAFVHAME GVIHAVGTSVP KLPHGLL
CAG35271.1 TGVDAFVHAME GVIHAVGTSVP KLPHGLL
WP_041277545.1 TGVDAFVHAME GVIHAVGTSVP KLPHGLL
OUO52689.1 TGIDAFVHAME GIIHAVGTAIP HMPHGVA
DhaT/CAD98878.1 TGMDALTHAVE GYVHAMAHQLG DMPHGVA
FucO/P0A9S1.2 TGVDALTHAIE GLVHGMAHPLG NTPHGVA

Fig. S8

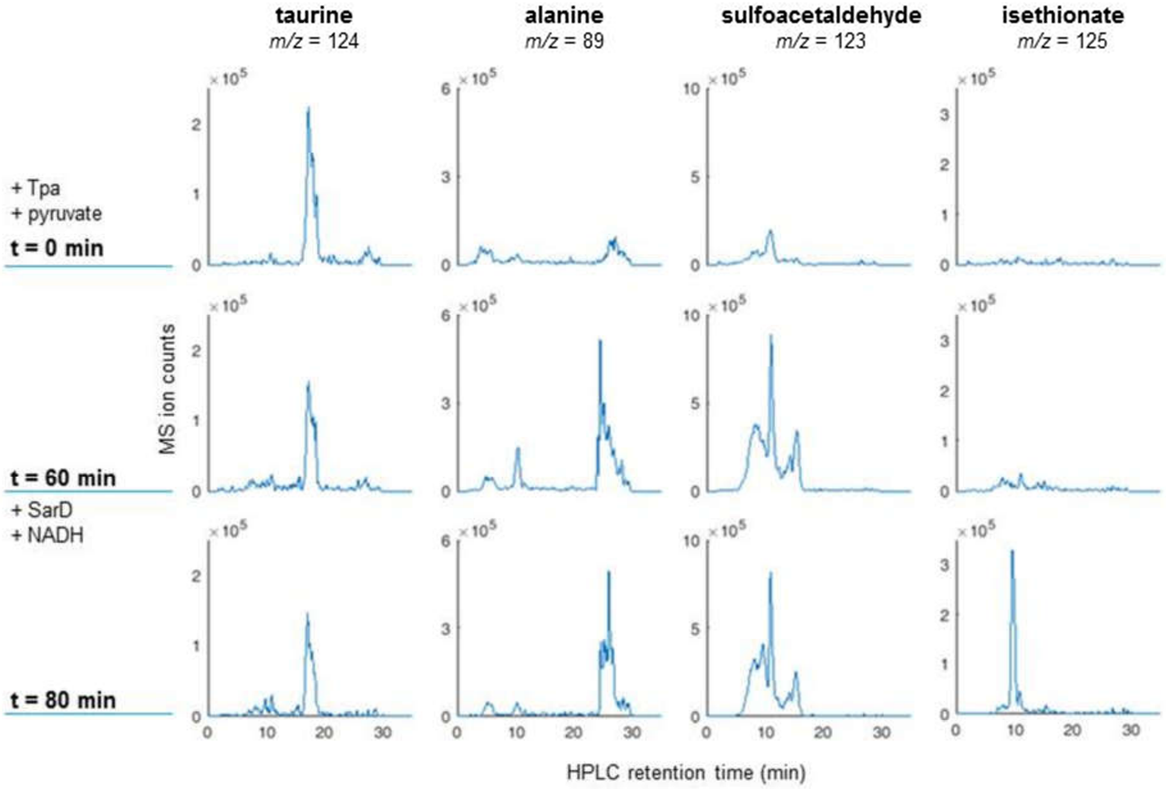
taurine
m/z = 124
alanine
m/z = 89
sulfoacetaldehyde
m/z = 123
isethionate
m/z = 125
+ Tpa
+ pyruvate
t = 0 min
t = 60 min
+ SarD
+ NADH
t = 80 min
MS ion counts
HPLC retention time (min)

Fig. S9

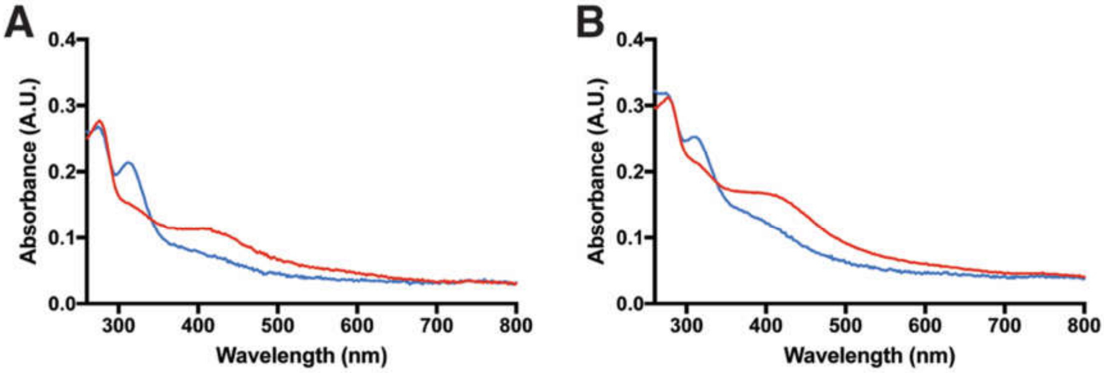

A
0.4
0.3
0.2
0.1
0.0
Absorbance (A.U.)
300
400
500
600
700
800
Wavelength (nm)
B
0.4
0.3
0.2
0.1
0.0
Absorbance (A.U.)
300
400
500
600
700
800
Wavelength (nm)

Fig. S10

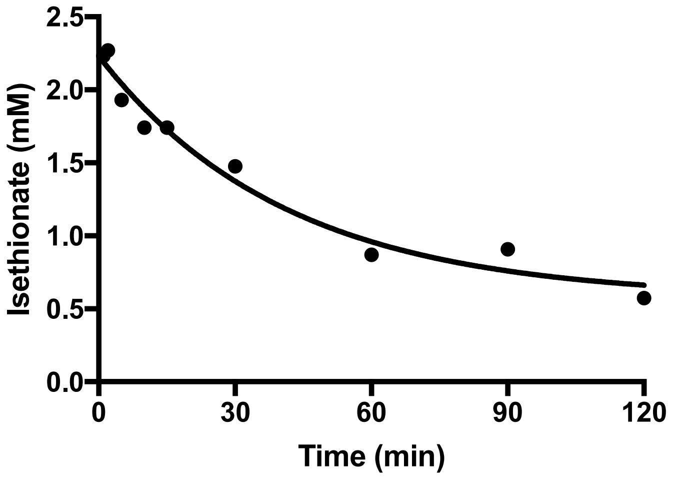

Fig. S11

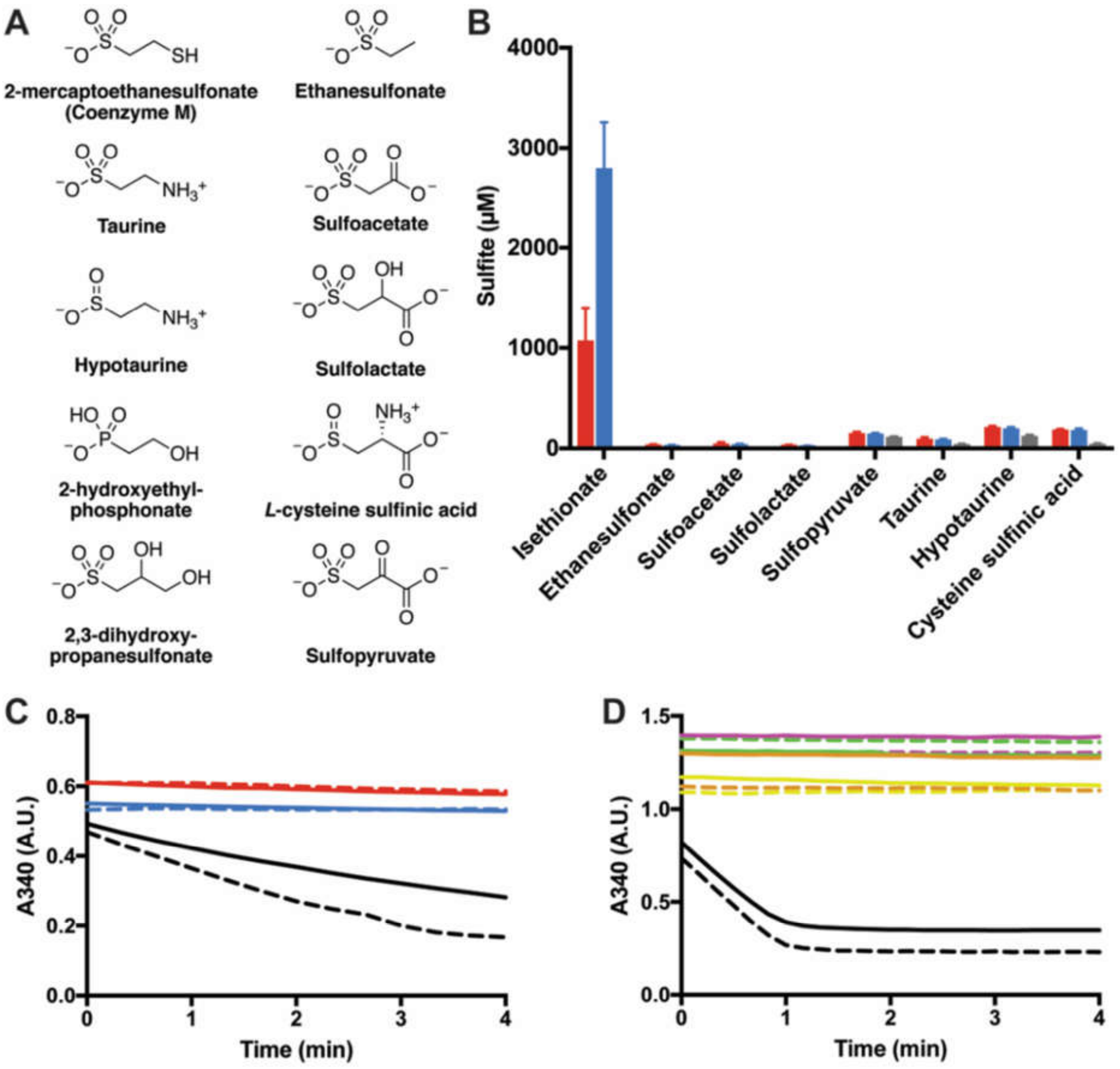
A
2-mercaptoethanesulfonate (Coenzyme M)
Ethanesulfonate
Taurine
Sulfoacetate
Hypotaurine
Sulfolactate
2-hydroxyethyl-phosphonate
L-cysteine sulfinic acid
2,3-dihydroxy-propanesulfonate
Sulfopyruvate
B
Sulfite (μM)
4000
3000
2000
1000
0
Isethionate
Ethanesulfonate
Sulfoacetate
Sulfolactate
Sulfopyruvate
Taurine
Hypotaurine
Cysteine sulfinic acid
C
A340 (A.U.)
Time (min)
D
A340 (A.U.)
Time (min)

Fig. S12

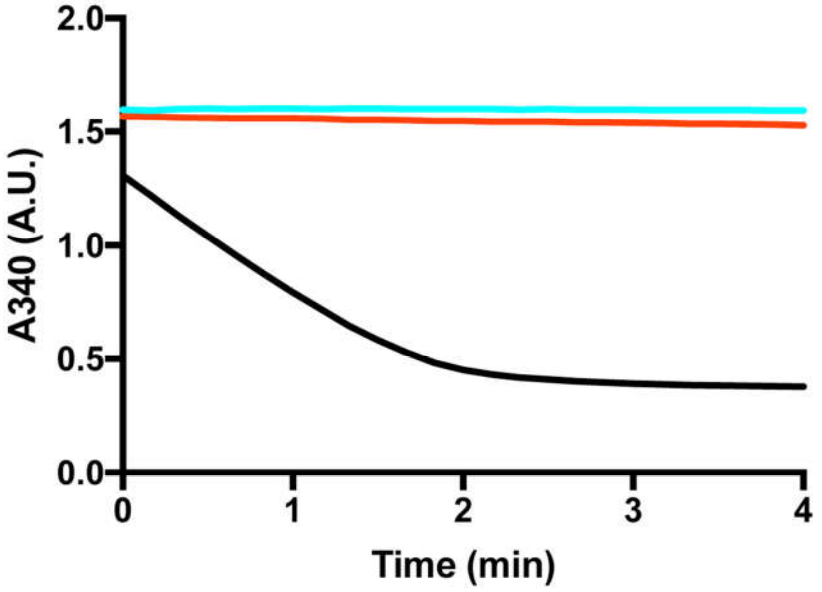

Fig. S13

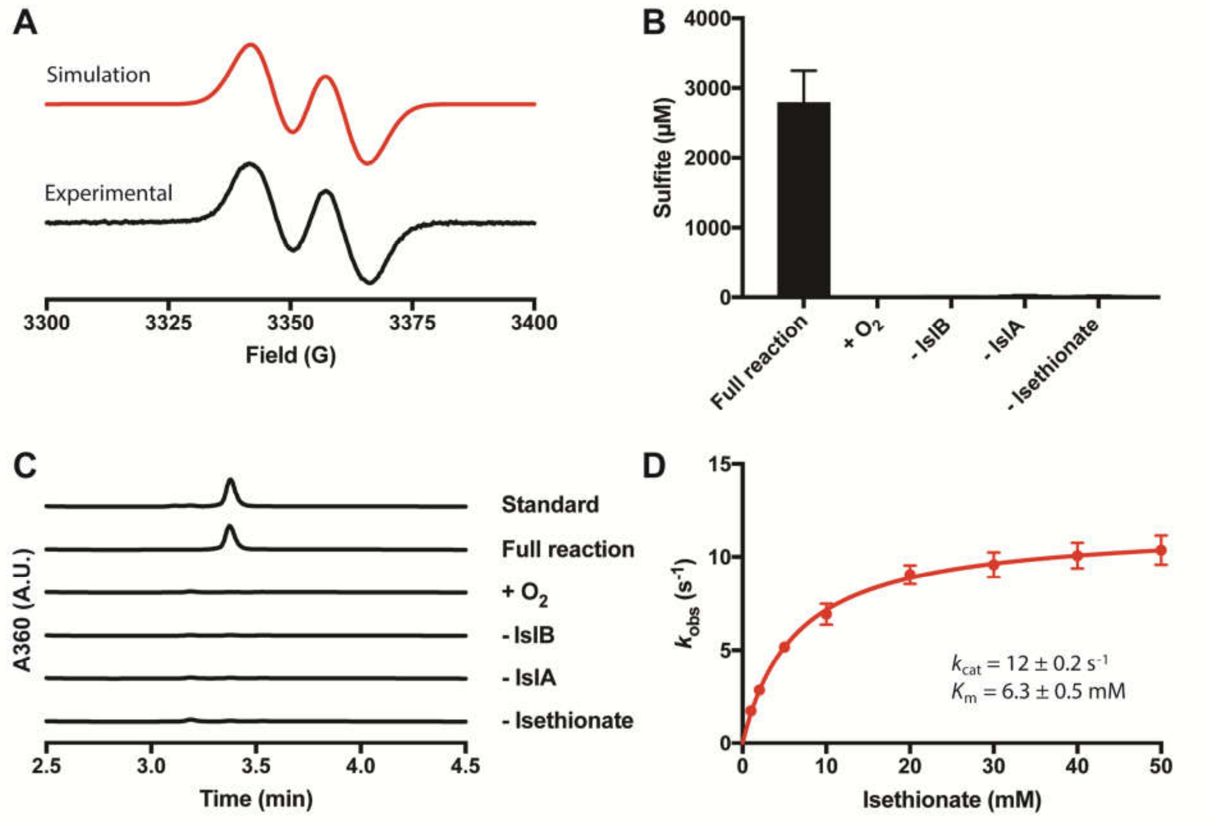
A
Simulation
Experimental
3300
3325
3350
3375
3400
Field (G)
B
4000
3000
2000
1000
0
Sulfite (µM)
Full reaction
+ O2
- IslB
- IslA
- Isethionate
C
Standard
Full reaction
+ O2
- IslB
- IslA
- Isethionate
A360 (A.U.)
2.5
3.0
3.5
4.0
4.5
Time (min)
D
15
10
5
0
kobs (s-1)
kcat = 12 ± 0.2 s-1
Km = 6.3 ± 0.5 mM
0
10
20
30
40
50
Isethionate (mM)

Fig. S14

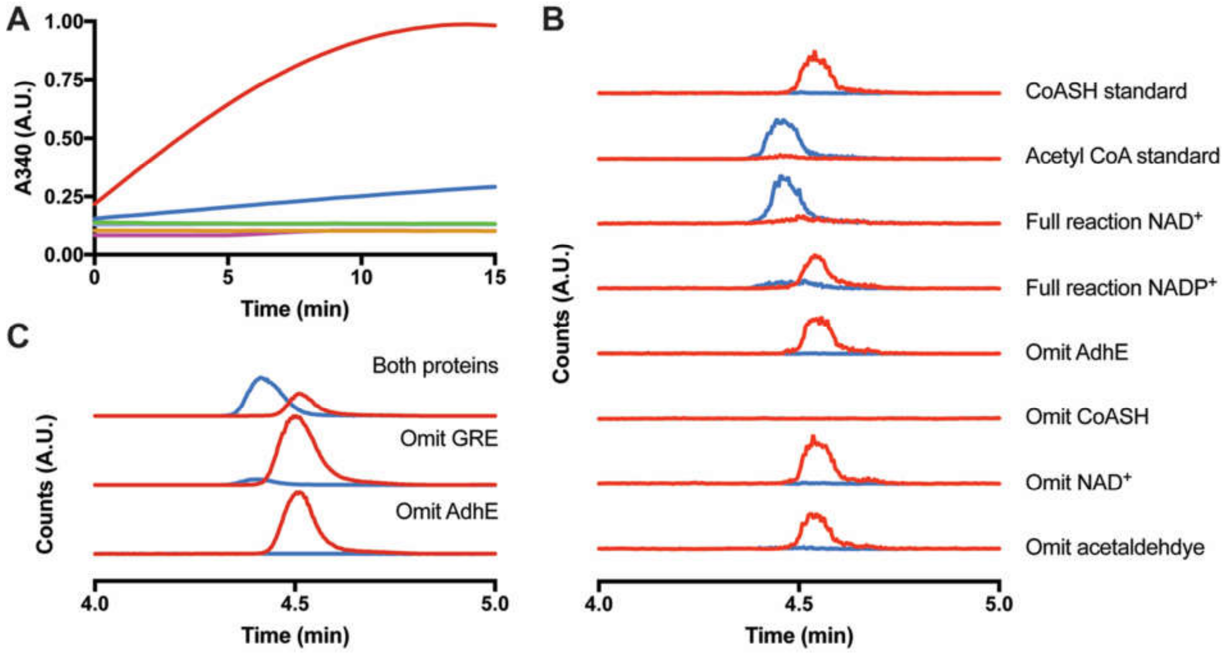
A
1.00
0.75
0.50
0.25
0.00
A340 (A.U.)
0
5
10
15
Time (min)
B
CoASH standard
Acetyl CoA standard
Full reaction NAD+
Full reaction NADP+
Omit AdhE
Omit CoASH
Omit NAD+
Omit acetaldehdye
Counts (A.U.)
4.0
4.5
5.0
Time (min)
C
Both proteins
Omit GRE
Omit AdhE
Counts (A.U.)
4.0
4.5
5.0
Time (min)

Fig. S15

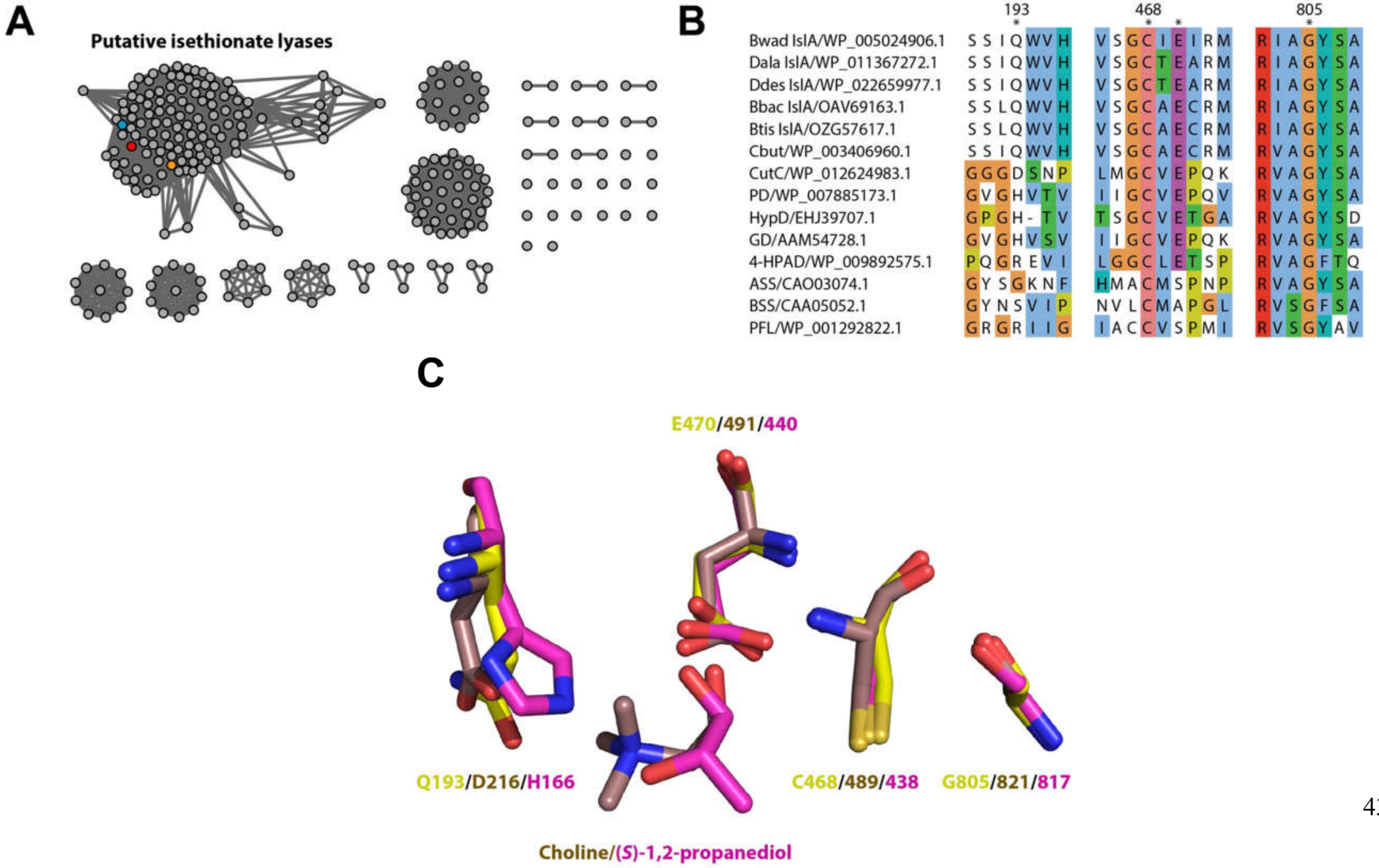

A
Putative isethionate lyases
B
193
468
805
Bwad IslA/WP_005024906.1 SSIQWVH VSGCIEIRM RIAGYSA
Dala IslA/WP_011367272.1 SSIQWVH VSGCTEARM RIAGYSA
Ddes IslA/WP_022659977.1 SSIQWVH VSGCTEARM RIAGYSA
Bbac IslA/OAV69163.1 SSLQWVH VSGCAECRM RIAGYSA
Btis IslA/OZG57617.1 SSLQWVH VSGCAECRM RIAGYSA
Cbut/WP_003406960.1 SSIQWVH VSGCAECRM RVAGYSA
CutC/WP_012624983.1 GGGDSNP LMGCVEPQK RVAGYSA
PD/WP_007885173.1 GVGHVTV IIGCVEPQV RVAGYSA
HypD/EHJ39707.1 GPGH-TV TSGCVETGA RVAGYSD
GD/AAM54728.1 GVGHVSV IIGCVEPQK RVAGYSA
4-HPAD/WP_009892575.1 PQGREVI LGGCLETSP RVAGFTQ
ASS/CAO03074.1 GYSGKNF HMACMSPNP RVAGYSA
BSS/CAA05052.1 GYNSVIP NVLCMAPGL RVSGFSA
PFL/WP_001292822.1 GRGRIIG IACCVSPMI RVSGYAV
C
E470/491/440
Q193/D216/H166
C468/489/438
G805/821/817
Choline/(S)-1,2-propanediol

Table S1. List of predicted isethionate sulfite-lyases or other C-S bond cleaving GREs as retrieved from the non-redundant NCBI database at a threshold of 62% identity.

No.	Annotation	Taxonomy					Accession
		Phylum	Class	Order	Family	Species / Organism	Genbank
1	Formate acetyltransferase	*Actinobacteria*	*Actinobacteria*	*Bifidobacteriales*	*Bifidobacteriaceae*	*Bifidobacterium callitrichos* DSM 23973	WP_043166045.1
2	Formate acetyltransferase	*Actinobacteria*	*Actinobacteria*	*Bifidobacteriales*	*Bifidobacteriaceae*	*Bifidobacterium tissieri*	OZG57617.1
3	Formate acetyltransferase	*Actinobacteria*	*Actinobacteria*	*Bifidobacteriales*	*Bifidobacteriaceae*	*Bifidobacterium tissieri*	WP_094663811.1
4	Glycyl radical enzyme	*Actinobacteria*	*Coriobacteriia*	*Eggerthellales*	*Eggerthellaceae*	*Gordonibacter sp.* An230	WP_087193818.1
5	4-Hydroxyphenylacetate decarboxylase	*Bacteroidetes*	*Bacteroidia*	*Bacteroidales*		*Bacteroidales* bacterium Barb6XT	OAV69163.1
6	Glycyl radical enzyme	*Bacteroidetes*	*Bacteroidia*	*Bacteroidales*		*Bacteroidales* bacterium Barb6XT	WP_066178498.1
7	Glycyl radical enzyme	*Firmicutes*	*Bacilli*	*Bacillales*	*Paenibacillaceae*	*Fontibacillus panacisegetis*	WP_091226859.1
8	Formate acetyltransferase	*Firmicutes*	*Bacilli*	*Bacillales*	*Paenibacillaceae*	*Paenibacillus borealis*	WP_042216496.1
9	Formate acetyltransferase	*Firmicutes*	*Clostridia*	*Clostridiales*	*Lachnospiraceae*	*Anaerostipes hadrus*	AQP40533.1
10	Formate acetyltransferase	*Firmicutes*	*Clostridia*	*Clostridiales*	*Lachnospiraceae*	*Anaerostipes hadrus* DSM 3319	EKY23466.1
11	Glycyl radical protein	*Firmicutes*	*Clostridia*	*Clostridiales*	*Clostridiaceae*	*Clostridium arbusti* SL206	WP_010235418.1
12	Formate acetyltransferase	*Firmicutes*	*Clostridia*	*Clostridiales*	*Clostridiaceae*	*Clostridium butyricum*	WP_027636278.1
13	Formate acetyltransferase	*Firmicutes*	*Clostridia*	*Clostridiales*	*Clostridiaceae*	*Clostridium butyricum*	WP_043853189.1
14	Glycyl radical enzyme	*Firmicutes*	*Clostridia*	*Clostridiales*	*Clostridiaceae*	*Clostridium butyricum*	WP_071982924.1
15	Formate acetyltransferase	*Firmicutes*	*Clostridia*	*Clostridiales*	*Clostridiaceae*	*Clostridium butyricum* E4 str. BoNT	WP_003406960.1
16	Benzylsuccinate synthase	*Firmicutes*	*Clostridia*	*Clostridiales*	*Clostridiaceae*	*Clostridium chromiireducens*	OPJ59679.1
17	Putative formate acetyltransferase	*Firmicutes*	*Clostridia*	*Clostridiales*	*Clostridiaceae*	*Clostridium cylindrosporum* DSM 605	WP_048570467.1
18	Pyruvate formate-lyase	*Firmicutes*	*Clostridia*	*Clostridiales*	*Clostridiaceae*	*Clostridium pasteurianum* BC1	WP_015614283.1
19	Glycyl radical protein	*Firmicutes*	*Clostridia*	*Clostridiales*	*Peptococcaceae*	*Dehalobacterium formicoaceticum*	WP_089608549.1
20	Glycyl radical enzyme	*Firmicutes*	*Clostridia*	*Clostridiales*	*Peptococcaceae*	*Desulfitibacter sp.* BRH_c19	KUO50820.1
21	Pyruvate formate-lyase	*Firmicutes*	*Clostridia*	*Clostridiales*	*Peptococcaceae*	*Desulfitobacterium dehalogenans*	WP_014792329.1

						ATCC 51507	
22	Formate acetyltransferase	*Firmicutes*	*Clostridia*	*Clostridiales*	*Peptococcaceae*	*Desulfitobacterium hafniense*	WP_011459062.1
23	Formate acetyltransferase	*Firmicutes*	*Clostridia*	*Clostridiales*	*Peptococcaceae*	*Desulfitobacterium hafniense* DCB-2	WP_015942724.1
24	Formate acetyltransferase	*Firmicutes*	*Clostridia*	*Clostridiales*	*Peptococcaceae*	*Desulfitobacterium hafniense* DP7	WP_005810281.1
25	Pyruvate formate-lyase	*Firmicutes*	*Clostridia*	*Clostridiales*	*Peptococcaceae*	*Desulfitobacterium hafniense* Y51	BAE82205.1
26	Pyruvate formate-lyase	*Firmicutes*	*Clostridia*	*Clostridiales*	*Peptococcaceae*	*Desulfosporosinus youngiae* DSM 17734	WP_007786432.1
27	Formate acetyltransferase	*Firmicutes*	*Clostridia*	*Clostridiales*	*Peptococcaceae*	*Desulfotomaculum aeronauticum* DSM 10349	WP_072912824.1
28	Glycyl radical protein	*Firmicutes*	*Clostridia*	*Clostridiales*	*Peptococcaceae*	*Desulfotomaculum arcticum* DSM 17038	WP_092474605.1
29	Glycyl radical enzyme	*Firmicutes*	*Clostridia*	*Clostridiales*	*Peptococcaceae*	*Desulfotomaculum ferrireducens*	AQS57885.1
30	Formate acetyltransferase	*Firmicutes*	*Clostridia*	*Clostridiales*	*Peptococcaceae*	*Desulfotomaculum kuznetsovii* DSM 6115	AEG16045.1
31	Glycyl radical protein	*Firmicutes*	*Clostridia*	*Clostridiales*	*Peptococcaceae*	*Desulfotomaculum nigrificans* DSM 574	WP_003540773.1
32	Formate acetyltransferase	*Firmicutes*	*Clostridia*	*Clostridiales*	*Peptococcaceae*	*Desulfotomaculum reducens* MI-1	WP_011879050.1
33	Formate acetyltransferase	*Firmicutes*	*Clostridia*	*Clostridiales*	*Peptococcaceae*	*Desulfotomaculum ruminis* DSM 2154	WP_013840536.1
34	Pyruvate formate-lyase	*Firmicutes*	*Clostridia*	*Clostridiales*	*Eubacteriaceae*	*Eubacterium cellulosolvens* 6	WP_004602188.1
35	Glycyl radical enzyme	*Firmicutes*	*Clostridia*	*Clostridiales*	*Eubacteriaceae*	*Eubacterium oxidoreducens*	WP_090171121.1
36	Glycyl radical enzyme	*Firmicutes*	*Clostridia*	*Clostridiales*	*Eubacteriaceae*	*Pseudobutyrivibrio sp.* OR37	WP_090549811.1
37	Glycyl radical enzyme	*Firmicutes*	*Clostridia*	*Clostridiales*		*Clostridiales* bacterium 36_14	OKZ78447.1
38	Formate acetyltransferase	*Firmicutes*	*Clostridia*	*Clostridiales*	*Lachnospiraceae*	*Lachnospiraceae* bacterium	SKB77358.1
39	Pyruvate formate-lyase	*Firmicutes*	*Clostridia*	*Clostridiales*	*Lachnospiraceae*	*Lachnospiraceae* bacterium 5_1_63FAA	EFV17604.1
40	Glycyl radical enzyme	*Firmicutes*	*Clostridia*	*Clostridiales*	*Peptococcaceae*	*Peptococcus niger*	WP_091791955.1
41	Formate acetyltransferase	*Firmicutes*	*Clostridia*	*Clostridiales*	*Lachnospiraceae*	*Pseudobutyrivibrio ruminis*	WP_074791333.1
42	Glycyl radical enzyme	*Firmicutes*	*Clostridia*	*Clostridiales*	*Lachnospiraceae*	*Pseudobutyrivibrio sp.* JW11	WP_090481662.1
43	Formate acetyltransferase	*Firmicutes*	*Clostridia*	*Clostridiales*	*Peptococcaceae*	*Peptococcaceae* bacterium BICA1-8	KJS86610.1
44	Pyruvate formate-lyase	*Firmicutes*	*Negativicutes*	*Selenomonadales*	*Selenomonadaceae*	*Anaerovibrio* sp. JC8	ORT99124.1

45	Formate acetyltransferase	*Firmicutes*	*Negativicutes*	*Selenomonadales*	*Selenomonadaceae*	*Anaerovibrio* sp. RM50	WP_027406814.1
46	Glycyl radical protein	*Firmicutes*	*Negativicutes*	*Selenomonadales*	*Sporomusaceae*	*Dendrosporobacter quercicolus* DSM 1736	WP_092073083.1
47	Glycyl radical protein	*Firmicutes*	*Negativicutes*	*Selenomonadales*	*Sporomusaceae*	*Propionispora vibrioides* DSM 13305	WP_091749809.1
48	Pyruvate formate-lyase	*Firmicutes*	*Negativicutes*	*Selenomonadales*	*Sporomusaceae*		SCM78628.1
49	Pyruvate formate-lyase	*Firmicutes*	*Negativicutes*	*Selenomonadales*	*Sporomusaceae*		SCM78658.1
50	Glycyl radical enzyme	*Firmicutes*	*Negativicutes*	*Selenomonadales*	*Sporomusaceae*	*Sporomusa silvacetica*	WP_094606507.1
51	Benzylsuccinate synthase	*Firmicutes*	*Negativicutes*	*Selenomonadales*	*Sporomusaceae*	*Sporomusa silvacetica* DSM 10669	OZC15302.1
52	Glycyl radical protein	*Firmicutes*	*Negativicutes*	*Selenomonadales*	*Sporomusaceae*	*Sporomusa silvacetica* DSM 10669	WP_094606471.1
53	Benzylsuccinate synthase	*Firmicutes*	*Negativicutes*	*Selenomonadales*	*Sporomusaceae*	*Sporomusa sphaeroides* DSM 2875	WP_075757473.1
54	Benzylsuccinate synthase	*Firmicutes*	*Negativicutes*	*Selenomonadales*	*Sporomusaceae*	*Sporomusa sphaeroides* DSM 2875	WP_075757489.1
55	Formate acetyltransferase	*Proteobacteria*	*Deltaproteobacteria*	*Desulfobacterales*	*Desulfobulbaceae*	*Desulfopila aestuarii* DSM 18488	WP_073616382.1
56	Glycyl radical protein	*Proteobacteria*	*Deltaproteobacteria*	*Desulfobacterales*	*Desulfobacteraceae*	*Desulfospira joergensenii* DSM 10085	WP_033398525.1
57	Formate acetyltransferase	*Proteobacteria*	*Deltaproteobacteria*	*Desulfobacterales*	*Desulfobulbaceae*	*Desulfotalea psychrophila* LSv54	WP_011190268.1
58	Uncharacterized protein	*Proteobacteria*	*Deltaproteobacteria*	*Desulfovibrionales*	*Desulfovibrionaceae*	*Bilophila sp.* 4_1_30	WP_009368175.1
59	Uncharacterized protein	*Proteobacteria*	*Deltaproteobacteria*	*Desulfovibrionales*	*Desulfovibrionaceae*	*Bilophila sp.* 4_1_30	WP_009733371.1
60	Glycyl radical protein	*Proteobacteria*	*Deltaproteobacteria*	*Desulfovibrionales*	*Desulfovibrionaceae*	*Bilophila wadsworthia*	WP_029436929.1
61	Glycyl radical enzyme	*Proteobacteria*	*Deltaproteobacteria*	*Desulfovibrionales*	*Desulfovibrionaceae*	*Bilophila wadsworthia*	WP_005027953.1
62	Glycyl radical protein	*Proteobacteria*	*Deltaproteobacteria*	*Desulfovibrionales*	*Desulfomicrobiaceae*	*Desulfomicrobium apsheronum*	WP_092373161.1
63	Formate acetyltransferase	*Proteobacteria*	*Deltaproteobacteria*	*Desulfovibrionales*	*Desulfomicrobiaceae*	*Desulfomicrobium baculatum* DSM 4028	WP_015773771.1
64	Formate acetyltransferase	*Proteobacteria*	*Deltaproteobacteria*	*Desulfovibrionales*	*Desulfomicrobiaceae*	*Desulfomicrobium baculatum* DSM 4028	WP_015774320.1
65	Glycyl radical protein	*Proteobacteria*	*Deltaproteobacteria*	*Desulfovibrionales*	*Desulfomicrobiaceae*	*Desulfomicrobium escambiense*	WP_028577862.1
66	Glycyl radical protein	*Proteobacteria*	*Deltaproteobacteria*	*Desulfovibrionales*	*Desulfomicrobiaceae*	*Desulfomicrobium norvegicum*	WP_092191323.1
67	Formate acetyltransferase	*Proteobacteria*	*Deltaproteobacteria*	*Desulfovibrionales*	*Desulfonatronaceae*	*Desulfonatronum thioautotrophicum*	WP_045218508.1
68	Formate acetyltransferase	*Proteobacteria*	*Deltaproteobacteria*	*Desulfovibrionales*	*Desulfomicrobiaceae*	*Desulfoplanes formicivorans*	WP_069857699.1
69	Formate acetyltransferase	*Proteobacteria*	*Deltaproteobacteria*	*Desulfovibrionales*	*Desulfomicrobiaceae*	*Desulfoplanes formicivorans*	WP_069858090.1

70	Formate acetyltransferase	*Proteobacteria*	*Deltaproteobacteria*	*Desulfovibrionales*	*Desulfovibrionaceae*	*Desulfovibrio bizertensis* DSM 18034	SKA70293.1
71	Glycyl radical protein	*Proteobacteria*	*Deltaproteobacteria*	*Desulfovibrionales*	*Desulfovibrionaceae*	*Desulfovibrio cuneatus* DSM 11391	WP_027187538.1
72	Formate acetyltransferase	*Proteobacteria*	*Deltaproteobacteria*	*Desulfovibrionales*	*Desulfovibrionaceae*	*Desulfovibrio desulfuricans*	SFW72879.1
73	Formate acetyltransferase	*Proteobacteria*	*Deltaproteobacteria*	*Desulfovibrionales*	*Desulfovibrionaceae*	*Desulfovibrio desulfuricans*	WP_020000116.1
74	Glycyl radical protein	*Proteobacteria*	*Deltaproteobacteria*	*Desulfovibrionales*	*Desulfovibrionaceae*	*Desulfovibrio desulfuricans*	WP_022658610.1
75	Glycyl radical protein	*Proteobacteria*	*Deltaproteobacteria*	*Desulfovibrionales*	*Desulfovibrionaceae*	*Desulfovibrio desulfuricans*	WP_041724859.1
76	Pyruvate formate-lyase	*Proteobacteria*	*Deltaproteobacteria*	*Desulfovibrionales*	*Desulfovibrionaceae*	*Desulfovibrio desulfuricans* ND132	WP_014322652.1
77	Formate acetyltransferase	*Proteobacteria*	*Deltaproteobacteria*	*Desulfovibrionales*	*Desulfovibrionaceae*	*Desulfovibrio desulfuricans subsp. desulf.* ATCC 27774	ACL49338.1
78	Glycyl radical enzyme	*Proteobacteria*	*Deltaproteobacteria*	*Desulfovibrionales*	*Desulfovibrionaceae*	*Desulfovibrio fairfieldensis*	AMD91514.1
79	Glycyl radical protein	*Proteobacteria*	*Deltaproteobacteria*	*Desulfovibrionales*	*Desulfovibrionaceae*	*Desulfovibrio fairfieldensis*	WP_083521970.1
80	Formate acetyltransferase	*Proteobacteria*	*Deltaproteobacteria*	*Desulfovibrionales*	*Desulfovibrionaceae*	*Desulfovibrio litoralis* DSM 11393	WP_072695368.1
81	Glycyl radical protein	*Proteobacteria*	*Deltaproteobacteria*	*Desulfovibrionales*	*Desulfovibrionaceae*	*Desulfovibrio oxyclinae* DSM 11498	WP_018124699.1
82	Formate acetyltransferase	*Proteobacteria*	*Deltaproteobacteria*	*Desulfovibrionales*	*Desulfovibrionaceae*	*Desulfovibrio piezophilus* C1TLV30	CCH48387.1
83	Glycyl radical protein	*Proteobacteria*	*Deltaproteobacteria*	*Desulfovibrionales*	*Desulfovibrionaceae*	*Desulfovibrio piezophilus* C1TLV30	WP_041720703.1
84	Pyruvate formate-lyase	*Proteobacteria*	*Deltaproteobacteria*	*Desulfovibrionales*	*Desulfovibrionaceae*	*Desulfovibrio piger*	WP_072335172.1
85	Formate acetyltransferase	*Proteobacteria*	*Deltaproteobacteria*	*Desulfovibrionales*	*Desulfovibrionaceae*	*Desulfovibrio piger* ATCC 29098	WP_006008826.1
86	Uncharacterized protein	*Proteobacteria*	*Deltaproteobacteria*	*Desulfovibrionales*	*Desulfovibrionaceae*	*Desulfovibrio sp.* 6_1_46AFAA	EGW52515.1
87	Glycyl radical protein	*Proteobacteria*	*Deltaproteobacteria*	*Desulfovibrionales*	*Desulfovibrionaceae*	*Desulfovibrio sp.* A2	EGY24021.1
88	Glycyl radical protein	*Proteobacteria*	*Deltaproteobacteria*	*Desulfovibrionales*	*Desulfovibrionaceae*	*Desulfovibrio sp.* A2	WP_007525054.1
89	Glycyl radical protein	*Proteobacteria*	*Deltaproteobacteria*	*Desulfovibrionales*	*Desulfovibrionaceae*	*Desulfovibrio sp.* A2	WP_043610275.1
90	Glycyl radical protein	*Proteobacteria*	*Deltaproteobacteria*	*Desulfovibrionales*	*Desulfovibrionaceae*	*Desulfovibrio sp.* An276	WP_087349870.1
91	Glycyl radical protein	*Proteobacteria*	*Deltaproteobacteria*	*Desulfovibrionales*	*Desulfovibrionaceae*	*Desulfovibrio sp.* An276	WP_087350898.1
92	Glycyl radical protein	*Proteobacteria*	*Deltaproteobacteria*	*Desulfovibrionales*	*Desulfovibrionaceae*	*Desulfovibrio sp.* An276	WP_087355015.1
93	Glycyl radical protein	*Proteobacteria*	*Deltaproteobacteria*	*Desulfovibrionales*	*Desulfovibrionaceae*	*Desulfovibrio sp.* MES5	OXS28002.1
94	Glycyl radical protein	*Proteobacteria*	*Deltaproteobacteria*	*Desulfovibrionales*	*Desulfovibrionaceae*	*Desulfovibrio termitidis*	WP_035067385.1
95	Glycyl radical protein	*Proteobacteria*	*Deltaproteobacteria*	*Desulfovibrionales*	*Desulfovibrionaceae*	*Desulfovibrio termitidis* HI1	WP_035063873.1

96	Formate acetyltransferase	*Proteobacteria*	*Deltaproteobacteria*	*Desulfovibrionales*	*Desulfovibrionaceae*	*Desulfovibrio vulgaris* DP4	WP_011791647.1
97	Pyruvate formate-lyase	*Proteobacteria*	*Deltaproteobacteria*	*Desulfovibrionales*	*Desulfovibrionaceae*	*Desulfovibrio vulgaris* RCH1	ADP87748.1
98	Formate acetyltransferase	*Proteobacteria*	*Deltaproteobacteria*	*Desulfovibrionales*	*Desulfovibrionaceae*	*Desulfovibrio vulgaris* str. Hildenborough ATCC 29579	WP_010940090.1
99	Glycyl radical enzyme	*Proteobacteria*	*Deltaproteobacteria*	*Desulfovibrionales*	*Desulfovibrionaceae*	uncultured *Desulfovibrio* sp.	SBV92506.1
100	Formate acetyltransferase	*Proteobacteria*	*Deltaproteobacteria*	*Desulfovibrionales*	*Desulfovibrionaceae*	uncultured *Desulfovibrio* sp.	SBW01768.1
101	Glycyl radical protein	*Proteobacteria*	*Deltaproteobacteria*	*Desulfovibrionales*	*Desulfovibrionaceae*	*Halodesulfovibrio aestuarii*	WP_027361990.1
102	Formate acetyltransferase	*Proteobacteria*	*Deltaproteobacteria*	*Desulfovibrionales*	*Desulfovibrionaceae*	*Halodesulfovibrio marinisediminis* DSM 17456	WP_074216573.1
103	Formate acetyltransferase	*Proteobacteria*	*Deltaproteobacteria*	*Desulfovibrionales*	*Desulfovibrionaceae*	*Halodesulfovibrio spirochaetisodalis*	WP_066852996.1
104	Glycyl radical enzyme	*Proteobacteria*	*Deltaproteobacteria*	*Desulfovibrionales*	*Desulfovibrionaceae*	*Mailhella massiliensis*	WP_077071615.1
105	Glycyl radical enzyme	*Proteobacteria*	*Deltaproteobacteria*	*Desulfovibrionales*	*Desulfovibrionaceae*	*Mailhella massiliensis*	WP_077073585.1
106	Glycyl radical protein	*Proteobacteria*	*Deltaproteobacteria*	*Desulfovibrionales*	*Desulfovibrionaceae*	*Mailhella massiliensis*	WP_077073949.1
107	Glycyl radical protein	*Proteobacteria*	*Deltaproteobacteria*	*Desulfovibrionales*	*Desulfovibrionaceae*	*Mailhella massiliensis*	WP_077073952.1
108	Formate acetyltransferase	*Proteobacteria*	*Deltaproteobacteria*	*Syntrophobacterales*	*Syntrophobacteraceae*	*Desulfacinum hydrothermale* DSM 13146	SMC22069.1
109	Formate acetyltransferase	*Proteobacteria*	*Deltaproteobacteria*	*Syntrophobacterales*	*Syntrophobacteraceae*	*Desulfacinum infernum* DSM 9756	WP_073041932.1
110	Pyruvate formate-lyase	*Proteobacteria*	*Gammaproteobacteria*	*Enterobacterales*	*Pectobacteriaceae*	*Brenneria goodwinii*	WP_048638610.1
111	Formate acetyltransferase	*Proteobacteria*	*Gammaproteobacteria*	*Enterobacterales*	*Pectobacteriaceae*	*Brenneria sp.* EniD312	WP_009112175.1
112	Glycyl radical protein	*Proteobacteria*	*Gammaproteobacteria*	*Enterobacterales*	*Pectobacteriaceae*	*Pectobacterium parmentieri* CFIA1002	WP_025920032.1
113	Formate acetyltransferase	*Proteobacteria*	*Gammaproteobacteria*	*Enterobacterales*	*Pectobacteriaceae*	*Pectobacterium parmentieri* WPP163	WP_015731066.1
114	Pyruvate formate-lyase	*Proteobacteria*	*Gammaproteobacteria*	*Enterobacterales*	*Pectobacteriaceae*	*Pectobacterium sp.* SCC3193	WP_014701133.1
115	Glycyl radical enzyme	*Proteobacteria*	*Gammaproteobacteria*	*Enterobacterales*	*Pectobacteriaceae*	*Pectobacterium wasabiae* CFBP 3304	WP_005975687.1

Table S2. List of PCR primers used.

IMG locus tag (gene)	forward primer	reverse complements primer
HMPREF0179_02714 (*B. wadsworthia* SarD)	*CGT*CATATGACTTACGATAAAGCTGAATTGGTCG	*ATT*CTCGAGAGTCCCGATGCCTCCGGATGAAGG
HMPREF0179_02713 (*B. wadsworthia* Tpa)	*CGT*CATATGGCTTTTGTGCATTACACCGTC	*ATT*CTCGAGTTGTTCAGGGCTTTTCTTTCGTTTTT
pET28a / HMPREF0179_00639 (*B. wadsworthia* IslA)	GCAGCGGCCTGGTGCCGCGCGGCAGCCATATGACTCAGGTAGCTGAAATCAAATC	GGATCTCAGTGGTGGTGGTGGTGGTGCTCGAGTTACATCTGGTCGTGGCCGGTACG
pET29b / HMPREF0179_00638 (*B. wadsworthia* IslB)	GTTTAACTTTAAGAAGGAGATATACATATGGGTTCCTTTGAAGATAGAAAGG	GATCTCAGTGGTGGTGGTGGTGGTGCTCGAGTGACTTCTTTTCTTCCGTCACGGC
pET29b / HMPREF0179_00640 (*B. wadsworthia* AdhE)	GTTTAACTTTAAGAAGGAGATATACATATGGACGTGCGCCAACAAGACG	GATCTCAGTGGTGGTGGTGGTGGTGCTCGAGTGAGACGATGCGGAAGTTGTCC
pET28a / G449DRAFT_2761 (*D. desulfuricans* IslA)	GCAGCGGCCTGGTGCCGCGCGGCAGCCATATGAGCACCACCACTTGCGAATGCC	GGATCTCAGTGGTGGTGGTGGTGGTGCTCGAGTTACATCACGTCGTGTTCAGTAC
pET29b / G449DRAFT_2762 (*D. desulfuricans* IslB)	GTTTAACTTTAAGAAGGAGATATACATATGTGCCTGGAAGACAGCCAACAGC	GATCTCAGTGGTGGTGGTGGTGGTGCTCGAGTGAGTGCGGTACGCGGAGCCGATC
D. desulfuricans DctP pET29b Fw	GTTTAACTTTAAGAAGGAGATATACATATGAGGCCGTCCAGGTTTATG	GATCTCAGTGGTGGTGGTGGTGGTGCTCGAGCGACTTCTGCGTTGCGGCGATCAG
pRL27_IE_rev1	ACTGAGAAGCCCTTAGAGCC	
D. alaskensis Δ1270	CCACCATGCTTATTCTGGGT	
D. alaskensis Δ1272	GGCATTTACCGAACCTGCTA	
D. alaskensis Δ1273	AGACATAGAAGAAGGCCGCA	
D. alaskensis Δ1274	CCTTCTACGGCCTCATCATC	
D. alaskensis Δ1275	CAGCAGGTGCATGCTGTAGT	
